# Shen-Zhi-Ling oral liquid ameliorates cerebral glucose metabolism disorder in early AD via insulin signal transduction pathway in vivo and in vitro

**DOI:** 10.1186/s13020-021-00540-0

**Published:** 2021-12-02

**Authors:** Gaofeng Qin, Yunfang Dong, Zhenhong Liu, Zhuoyan Gong, Chenyan Gao, Mingcui Zheng, Meijing Tian, Yannan He, Liqun Zhong, Pengwen Wang

**Affiliations:** 1 Key Laboratory of Chinese Internal Medicine of Ministry of Education and Beijing, Dongzhimen Hospital, Beijing University of Chinese Medicine (BUCM), Haiyuncang No. 5 in Dongcheng District, Beijing, China; 2 Binzhou Medical University Hospital, Shandong, China; 3 Institute for Brain Disorders, Beijing University of Chinese Medicine (BUCM), Beijing, China; 4 Beijing Prominion Publishing Co. Ltd, Beijing, China

**Keywords:** Shenzhiling oral liquid, Glucose metabolism, Glucose transporter, Glucolysis, SH-SY5Y cells, Insulin signal transduction

## Abstract

**Background:**

Shen-Zhi-Ling oral liquid (SZL) is an herbal formula known for its efficacy of nourishing “heart and spleen”, and is used for the treatment and prevention of middle- and early-stage dementia. This study investigated the effects of SZL on amelioration of AD, and examined whether the underlying mechanisms from the perspective of neuroprotection are related to brain glucose metabolism.

**Methods:**

Firstly, LC–MS/MS was used to analysis the SZL mainly enters the blood component. Then, the effects of SZL on cognitive and behavioral ability of APP/PS1 double transgenic mice and amyloid protein characteristic pathological changes were investigated by behavioral study and morphological observation. The effects of SZL on the ultrastructure of mitochondria, astrocytes, and micrangium related to cerebral glucose metabolism were observed using transmission electron microscopy. Then, micro-PET was also used to observe the effects of SZL on glucose uptake. Furthermore, the effects of SZL on insulin signaling pathway InR/PI3K/Akt and glucose transporters (GLUT1 and GLUT3) were observed by immunohistochemistry, Western-blot and RT-qPCR. Finally, the effects of SZL on brain glucose metabolism and key enzyme were observed. In vitro, the use of PI3K and/or GSK3β inhibitor to observe the effects of SZL drug-containing serum on GLUT1 and GLUT3.

**Results:**

In vivo, SZL could significantly ameliorate cognitive deficits, retarded the pathological damage, including neuronal degeneration, Aβ peptide aggregation, and ultrastructural damage of hippocampal neurons, improve the glucose uptake, transporters and glucolysis. Beyond that, SZL regulates the insulin signal transduction pathway the insulin signal transduction pathway InR/PI3K/Akt. Furthermore, 15% SZL drug-containing serum increased Aβ_42_-induced insulin signal transduction-pathway related indicators and GLUT1 and GLUT3 expression in SH-SY5Y cells. The improvement of GLUT1 and GLUT3 in the downstream PI3K/Akt/GSK3β signaling pathway was reversed by the use of PI3K and/or GSK3β inhibitor.

**Conclusions:**

In summary, our results demonstrated that improving glucose uptake, transport, and glycolysis in the brain may underlie the neuroprotective effects of SZL, and its potential molecular mechanism may be related to regulate the insulin signal transduction pathway.

**Supplementary Information:**

The online version contains supplementary material available at 10.1186/s13020-021-00540-0.

## Introduction

Alzheimer’s disease (AD), the most prevalent form of dementia, is characterized by beta-amyloid (Aβ) plaque deposition and neurofibrillary tangles of hyperphosphorylated tau protein [1]. The pathogenesis of AD is complex. While it has been found that administration of compounds and natural products that target Aβ and tau proteins can improve the cognitive function of AD model animals, the development and clinical research of new AD drugs is still lacking. According to recent research, the initial events of AD deserve more attention [2]. For example, impairment of brain glucose metabolism is a pathophysiological feature of AD that occurs before cognitive dysfunction and pathological changes [3, 4].

Hyperinsulinemia and insulin resistance are not only pathophysiological characteristics of Type 2 Diabetes Mellitus (T2DM), but also have a significant impact on cognitive dysfunction [5]. AD has been increasingly recognized as a metabolic disease, and the close relationship between AD and T2DM has attracted increasing attention from researchers. The risk factors, comorbidities, and pathophysiological mechanisms of AD and T2DM overlap greatly [3]. Common pathophysiological characteristics of AD and T2DM include insulin signal transduction disorders, cerebral microvascular lesions, inflammation, and oxidative stress [2, 6]. Studies have shown that the imbalance of glucose metabolism homeostasis in the brain, which is closely related to defects in the insulin signaling pathway Insulin-like receptor/phosphoinositide 3-kinase/protein kinase B (InR/PI3K/Akt), plays an important role in the early onset of AD [3, 4]. The mechanisms underlying the characteristic pathological changes of Aβ plaques and neurofibrillary tangles in early AD is still unclear. Some research has suggested that changes in cerebral glucose metabolism is the upstream trigger factor [7]. Emerging evidence has also indicated that insulin influences cerebral bioenergetics, enhances synaptic viability and dendritic spine formation, and clears Aβ peptides and phosphorylation of tau [8].

The use of Traditional Chinese Medicine in AD treatment has increasingly supported, with advantages of multi-target action, mild side effects, and good safety and reliability. The complex etiology and pathogenesis of AD coincide with the multi-target action of Traditional Chinese Medicine. Shen-Zhi-Ling oral liquid (SZL) is a traditional Chinese preparation approved by the China Food and Drug Administration that is currently used for the treatment or prevention of mild-to-moderate AD, given its efficacy in nourishing the “heart and spleen”. SZL is composed of Codonopsis pilosula, Cassia Twig, Radix Paeoniae Alba, Glycyrrhizae Radix (processed with honey), Poria, Zingiberis rhizoma, Cortex et Radix Polygalae, Rhizoma acori graminei, Os Draconis, and Concha Ostreae [9].

**Fig. 1:**
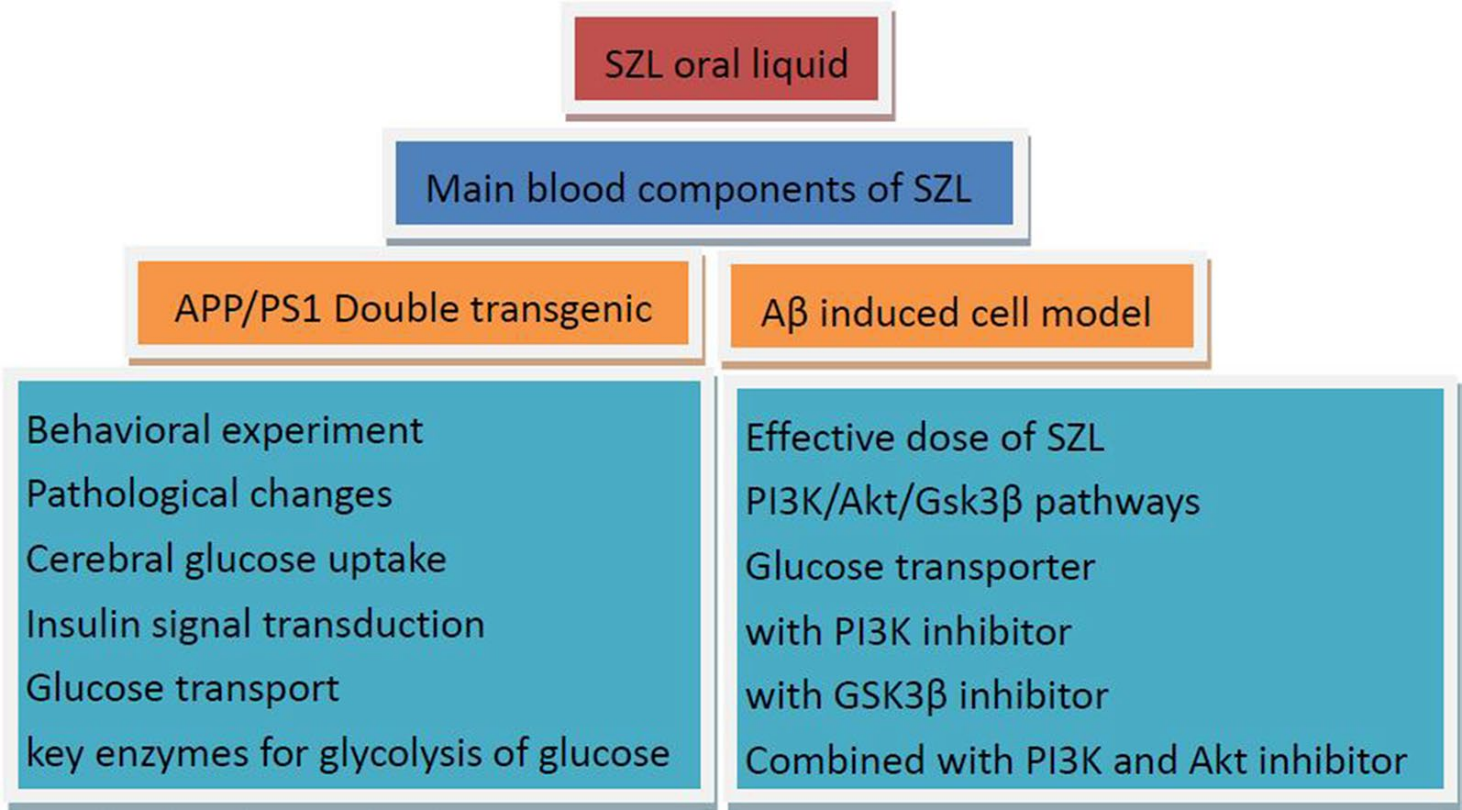
The research framework of the study

SZL has been found to improve learning and memory ability in APP/PS1 transgenic mice [10, 11]. Moreover, our previous findings have indicated that SZL exerts a neuroprotective effect on the myelin sheath, and that its mechanism of action is closely associated with activation of the PI3K/Akt/mTOR signaling pathway in APP/PS1 transgenic mice [12]. Based on these considerations, we hypothesized that the ability of SZL to reinforce and improve learning and memory ability in AD is associated with counteracting the imbalance of brain energy metabolism. The research framework of the study is shown in Fig. 1.

## Materials and methods

### Animals and drug administration

**Fig. 2:**
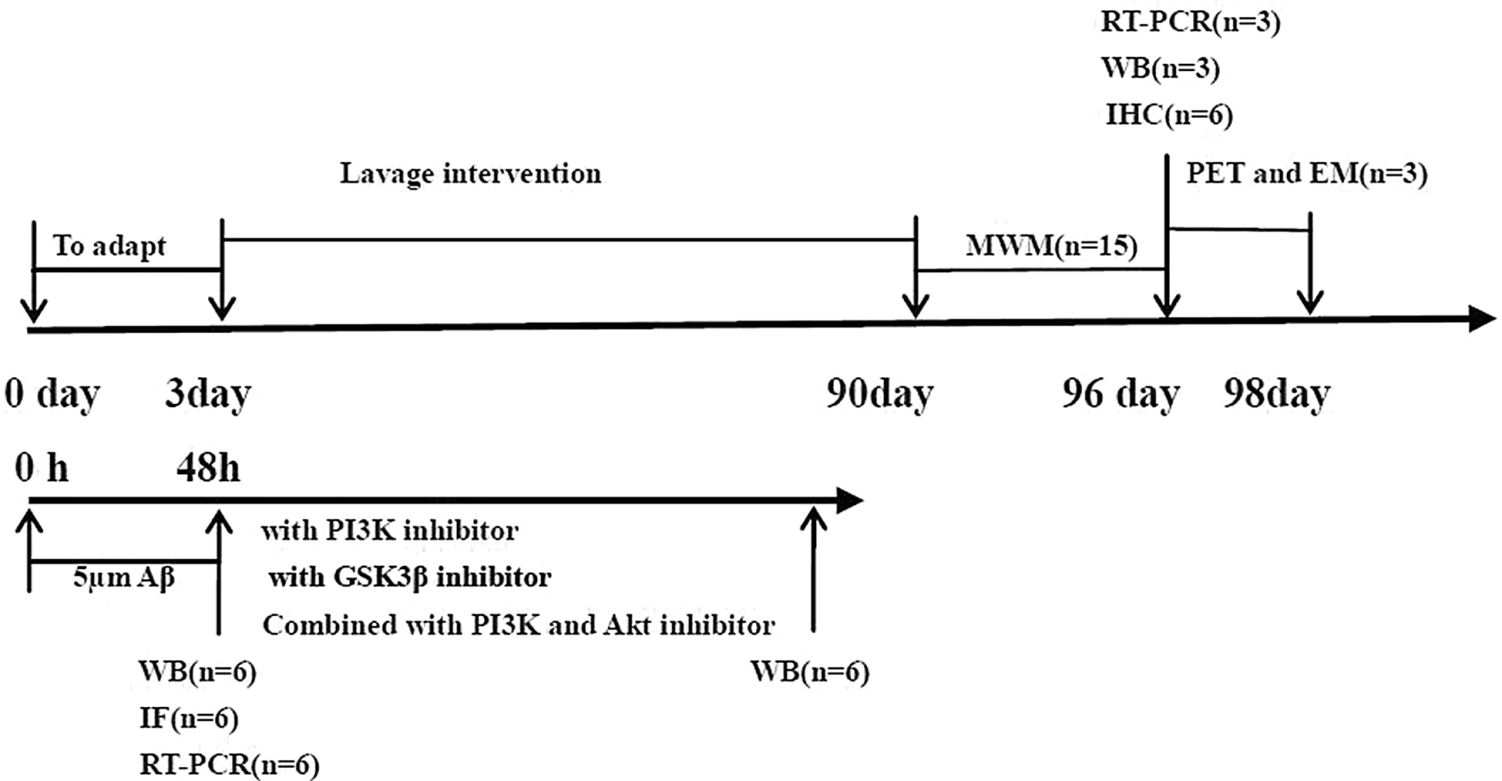
Experimental program

A total of fifteen 3-month-old male C57BL/6 J wild-type mice and forty-five 3-month-old male APP/PS1 double transgenic mice with the body weight of 25 ± 5 g were purchased from Beijing Huafukang Experimental Animal Technology Co., Ltd. (Beijing, China), with the license [SCXK(Beijing)2019–0008]. All mice were given 0.1 ml/10 g by gavage for 3 months after a week of adjustable feeding, and the dose of different groups of mice was converted according to the same proportion of adult dose. Donepezil group was given donepezil (0.92 mg/kg/d), and SZL group was given SZL (2.9 ml/kg/d) by intragastric administration with 0.5% CMC. Control group and model group were given equal volume of 0.5% CMC. Thirty male adult SD rats with the body weight of 200 ± 20 g were purchased from Beijing Vitong Lihua Experimental Animal Technology Co., Ltd. [SCYK(Beijing)2016-0006]. Rats were randomly divided into control group and SZL group, and fed for three days. SZL group was given gavage twice the equivalent dose of adult (70 kg), and the control group was given gavage of 0.5% CMC, once a day, for consecutive 7 days. Two hours after the last administration, sterile abdominal aorta blood was collected for serum separation. The serum was inactivated at 56 ℃ for 30 min, filtered with 0.22 μm filter membrane, divided into different packages, and frozen at – 80 ℃. Figure 2 presents a schematic timeline of animal and cell experiments. All mice and rats were reared in the Barrier Environmental Animal Room of Dongzhimen Hospital Chinese Medicine Pharmacology Laboratory, license [SXXK(Beijing)2015-0001, 2020-0013]. Animal experiments have been approved by Dongzhimen Hospital Experimental Animal Ethics Committee, Ethics Number: 19-19, 20-50. All mice were gavaged daily started with the first administration and continued until the end of the study. SZL, Shandong Wohua Pharmaceutical Co., Ltd., National license Z20120010. Preparation and analysis were the same as those employed in our previous study [12].

### Morris Water Maze

Morris water maze experiment can be divided into directional navigation experiment and space exploration experiment. The whole experiment lasts for 6 days, including 5 days directional navigation experiment (the ability to acquire learning and memory) and 1 day space exploration experiment (the ability to explore space). The specific steps are the same as in our previous experiment [9].

### Micro-positron emission tomography imaging

Cerebral extraction and accumulation of glucose was scanned using a micro-positron emission tomography (micro-PET) imaging system (Inveon PET/CT, Siemens Healthcare, Erlangen, Germany) [13]. Three mice were randomly selected from each group and fasted for 6 h before the experiment. Mice were placed in transparent boxes and completely anesthetized by inhalation of 2% isoflurane. Radioactive tracer ^18^F-FDG of approximately 530–650 Ci was administered through the tail vein. Micro-PET images were collected 1 h after injection. The whole brain three-dimensional region of interest was manually selected from cross-sectional, sagittal, and coronal PET/CT images of mice to calculate the percentage of dose per gram of brain tissue injected in the region of interest (%ID/g).

### Transmission electron microscopy

Three mice from each group were anesthetized using tribromoethanol after the last behavioral test [12]. Neurons in the hippocampal CA1 region of each group were observed under a × 1.5 k magnification. The neuronal organelles in the hippocampal CA1 region of mice in each group were observed under a × 8.0 k magnification. The micrangium in the hippocampal CA1 region of mice were observed under a × 4.0 k magnification. Astrocytes in the hippocampal CA1 region of mice in each group were observed under a × 2.0 k magnification.

### Hematoxylin and Eosin (HE)and Immunohistochemical staining

The brain tissues of 3 mice from each group were collected and fixed with 4% paraformaldehyde, embedded in paraffin, and sectioned at a thickness of 4 μm. The oven was heated to 56 °C, and the selected sections were baked for 1 h. The brain sections were deparaffinized in xylene and rehydrated. For staining, differentiation, and antiblue staining with hematoxylin, the sections were immersed in hematoxylin for 3 min, and then differentiated with 1% hydrochloric acid for 10 s. After eosin had been impregnated for 3 min, the sections were then dehydrated by gradient. Finally, the film was sealed and photographed under a microscope. The immunohistochemical assay procedure was the same as that of our previous experiment [9, 12]. The brain slices was incubated with the following primary antibodies: Aβ_42_ (1:1500, ab201060, Abcam, USA), InR (1: 500, ab5500, Abcam, USA), IRS2 (1:500, ab134101, Abcam, USA), GSK3β (1:500, ab32391, Abcam, USA), p-GSK3β (1:500, ab75814, Abcam, USA). The slices were observed under a × 20 objective lens of an optical microscope. The Aβ plaques was observed under a × 5 objective lens of an optical microscope.

### Cell culture, Aβ_42_ preparation

SH-SY5Y cells were purchased from China Academy of Chinese Medical Sciences. SH-SY5Y cells were cultured in high-glucose Dulbecco’s Modified Eagle’s Medium (DMEM) supplemented with 10% fetal bovine serum. The logarithmically grown cells were inoculated in a 25-cm cell culture flask at a density of 2000 cells/ml at 37 °C in a CO_2_ incubator for 48 h. The molecular weight of Aβ_42_ was 4515.14 g/mol. Soluble 1 mg Aβ_42_ was dissolved in 22.15 μl dimethyl sulfoxide, and 22.15 ml DMEM was added to prepare the mother liquor of 10 μm. The cells were left in an incubator at 37 °C CO_2_ incubators for 7 days. Cells were incubated with 5 μM Aβ_42_ for 48 h as model, and the specific modeling method and model identification was shown in Additional file 1.

### Immunofluorescence

Cells were seeded on a 24 well Glass slide for 48 h and washed 3 times by PBS and then fixed with 10% paraformaldehyde for 40 min, using 0.1% Tritonx-100 appear on the ice for 10 min. Cells incubated by 5% goat serum for 1 h at room temperature, and the primary antibody against GLUT1 (1:500) overnight at 4℃, then washed 3 times by PBS and incubated by the secondary antibody (Alexa flour® 488 Goat anti-rabbit IgG) for 2 h at room temperature. The nuclei were stained by DAPI. The mean fluorescence intensities of GLUT1 in 6 fields of view per group were calculated for statistical analysis by Image J.

### Western blot analysis

Protein lysates were prepared by freezing hippocampal tissues or cell homogenates with RIPA lysates containing proteases and phosphatase inhibi tors. After centrifuged at 12,000 rpm for 15 min at 4 °C, the supernatant was collected. Protein concentrations were measured by BCA kit (Bioss, China). The proteins were loaded and separated by SDS-PAGE and then transferred to PVDF membrane. The membrane was blocked by 5% defatted milk for 1 h. The membrane was then incubated with the following primary antibodies: Aβ_42_ (1:1500, ab201060, Abcam, USA), InR (1:1000, ab5500, Abcam, USA), p-InR (1:1000, ab60946, Abcam, USA), IRS2 (1:1000, ab134101, Abcam, USA), p-IRS2 (1:1000, ab3690, Abcam,USA),PI3K (1:1000, ab151549, Abcam, USA), Akt (1:1000, ab179463, Abcam, USA), p-Akt (1:1000, ab8805, Abcam, USA), GSK3β (1:1000, ab32391, Abcam, USA), p-GSK3β (1:1000, ab75814, Abcam, USA), GLUT1 (1:1000, ab652, Abcam, USA), GLUT3 (1:1000, ab41525, Abcam, USA),and β-actin (1:10,000, AY0573, Abways, China) at 4 °C overnight. After rinsing, the membrane was probed with secondary antibody at room temperature for 1 h. Finally, enhanced chemiluminescence reagent detection system was used to visualize the protein expression. Protein blots were quantified using ImageJ software and results were expressed quantitatively after normalizing blots with β-actin.

### Real-time PCR

Total RNA was extracted from the hippocampus or SH-SY5Y cells using Trizol reagent, and RNA was purified using chloroform and isopropanol. RNA was converted into cDNA using superscript reverse transcriptase and Taq polymerase by reverse transcription polymerase chain reaction (RT-PCR). Glyceraldehyde 3-phosphate dehydrogenase was used as an international control in the hippocampus, and β-actin was used as an international control in SH-SY5Y cells. Relative mRNA expressions were calculated using the 2^−ΔΔ^CT method [13]. RT-PCR was performed using the hippocampus primers shown in Table 1, and the SH-SY5Y cells in Table 2.

**Table 1:** The primer sequence of hippocampus

Name	Forward	Reverse	Size (bp)
InR	TGGCATGGCATACTTGAACG	AGGTGACATCCACCTCACAG	181 bp
IRS2	GGGCGAACTCTATGGGTACA	CTAGAGAGGCAGAGGAAGGC	179 bp
GSK	GATTCAGGCCGCTGCTTCAC	GGTCTGTCCACGGTCTCCAG	76 bp
GLUT1	AGAGGGTCGGCAGATGATGC	ACCACAGCGATGAGGATGGG	93 bp
HK-1	ACCAACCCACAAAACAACGC	CCAAGGAAACACCACTCCGA	112 bp
COXIV	GCCTTGGACGGCGGAATG	AGCGTAAGTGGGGAAAGCAT	132 bp
ATPase	CTGGCTGAGAACGGTTTCCT	AACGCTGTATGGCAGGTGAA	149 bp
AMPK	GAAAGTGAAGGTGGGCAAGC	GATGTGAGGGTGCCTGAACA	145 bp
GAPDH	TGCCCCCATGTTTGTGATG	TGTGGTCATGAGCCCTTCC	151 bp

**Table 2:** The primer sequence of SH-SY5Y cells

Name	Forward	Reverse	Size (bp)
PI3K	AAGTGCCAGAGTGAAGTGGC	ACTCCCCCTTCCCAAAGCTA	137 bp
Akt	ACTGTCATCGAACGCACCTT	CTCCTCCTCCTCCTGCTTCT	108 bp
GSK3β	CGAGACACACCTGCACTCTT	TTAGCATCTGACGCTGCTGT	143 bp
GLUT1	GGCTTCTCCAACTGGACCTC	CCGGAAGCGATCTCATCGAA	176 bp
GLUT3	GCACATAGCTATCAAGTGTGCTTT	CCTGCCTTACTGCCAACCTA	97 bp
β-actin	CTCCATCCTGGCCTCGCTGT	GCTGTCACCTTCACCTTTCC	268 bp

### Statistical analysis

Statistical software SPSS v. 20 was used for data analysis. Data graphs were drawn using GraphPad Prism 8 software. All data were first tested for normal distribution and homogeneity of variance. One-way analysis of variance (ANOVA) was used for comparisons between groups. The least significant difference test was used to assess homogeneity of variances, and Dunnett’s T3 test was used to assess heterogeneity of variances. Data are described as the mean ± standard deviation ($\overline{\text{x}}$ ± s). *P* < 0.05 was considered statistically significant, and *P* < 0.01 was more significant.

## Results

### Qualitative analysis of bioactive compounds in SZL

**Fig. 3:**
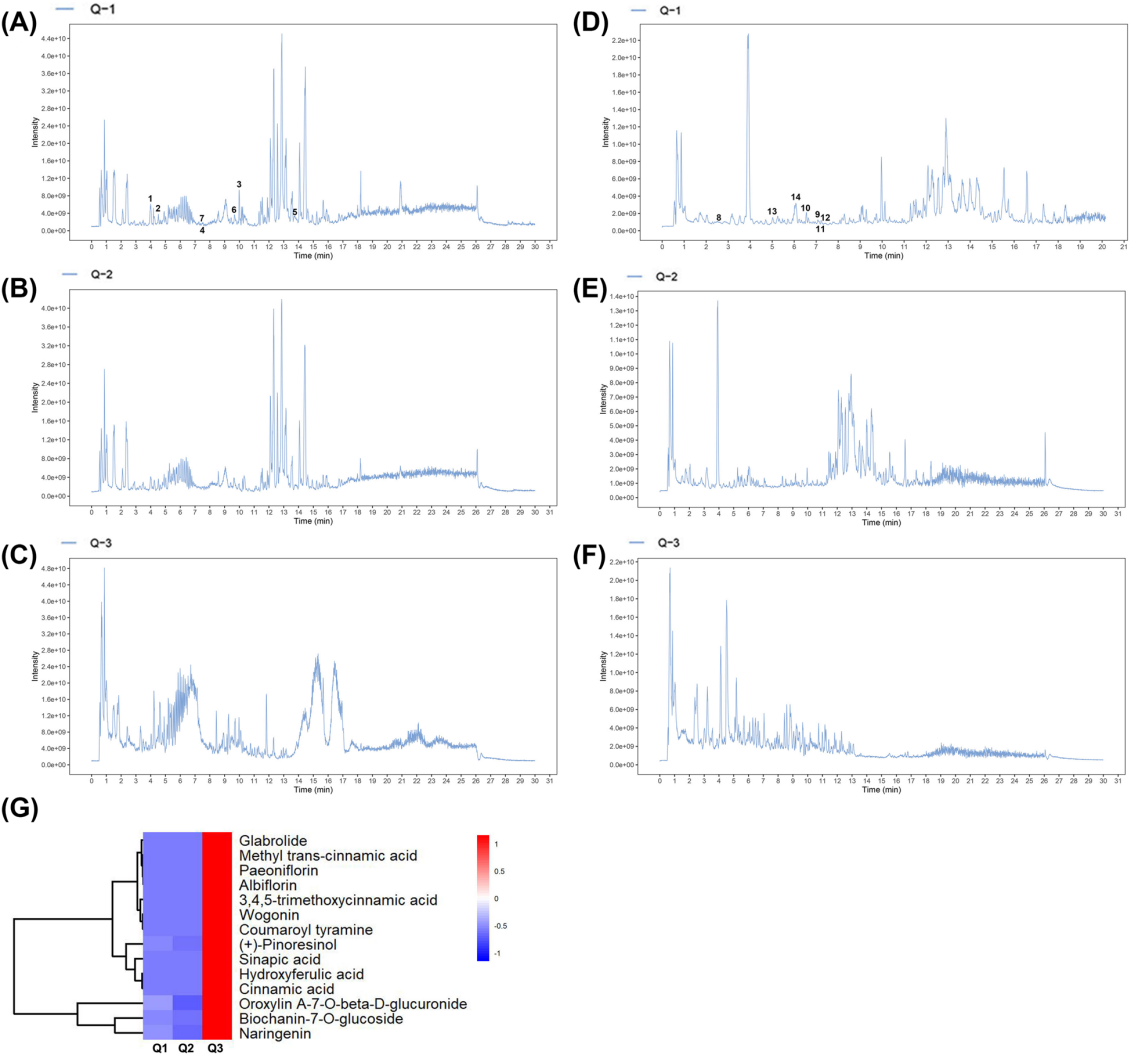
LC–MS/MS was used to analyze the main blood components of SZL. Total ion flow pattern in positive ion mode was detected by UHPLC-QE-MS (**A**–**C**). Total ion flow pattern in negative ion mode was detected by UHPLC-QE-MS (**D**–**F**). SZL serum containing drug (**A**, **D**), Blank serum without drug (**B**, **E**), SZL oral solution (**C**, **F**). Heat map of the main components of SZL into blood (**G**)

Liquid chromatography with tandem-mass spectrometry (LC–MS/MS) was used to identify the active components of SZL and SZL-containing serum, which identified 46 SZL components. The main components of Codonopsis pilosula, which tonifies qi spleen, Paeonia lactiflora, which nourishes the blood and promotes blood circulation, Radix Polygalae, which reduces phlegm and initiates orifices, and calamus can all enter the blood (Fig. 3). SZL mainly enters the blood component was shown in Additional file 2.

### SZL ameliorated cognitive deficits in APP/PS1 double transgenic mice

**Fig. 4:**
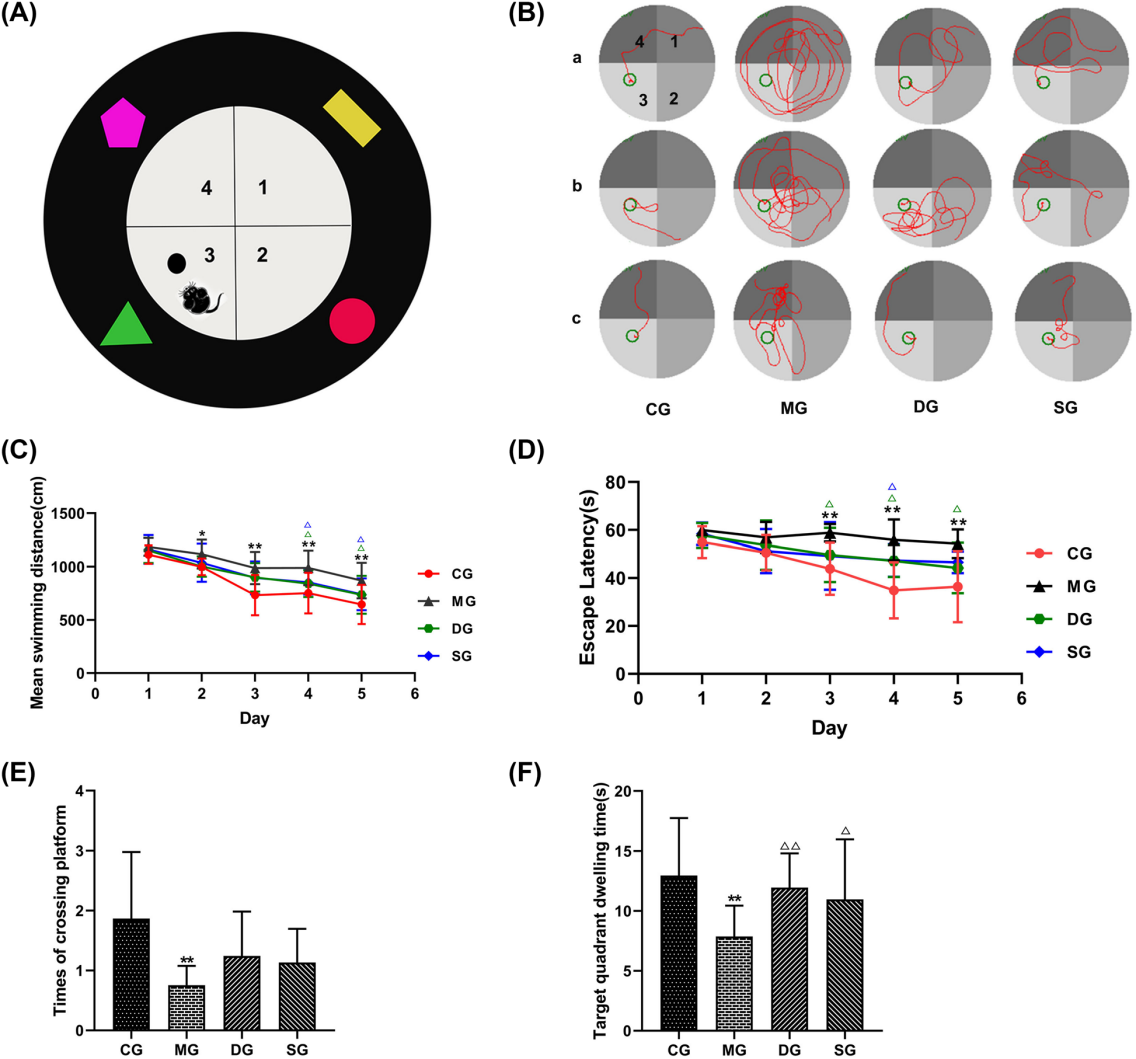
Effect of SZL cognitive deficits and improved memory abilities in APP/PS1 mice. Simple pattern of MWM (**A**). The figures represent the first, second, third, and fourth quadrants, respectively. Each quadrant of the inner wall of the MWM has visual signs to help the mice remember route. The platform is in the third quadrant effect of SZL treatment on the moving track (**B**). The movement trajectories of mice in each group entering water in different quadrants on the fifth day were selected for comparison. The first quadrant is in the upper left corner of the moving track map, and the remaining quadrants are 2, 3, and 4 in the counterclockwise direction. The effect of SZL treatment on mean swimming distances (**C**), Escape latency (**D**) times of crossing platform (**E**) and target quadrant dwelling time (**F**) in the MWM test. CG: control group, MG: model group, DG: donepezil group, SG: SZL group. All data are presented as means ± SEM (*n* = 15). *P < 0.05, **P < 0.01 versus Control group, ^**△**^*P* < 0.05, ^**△△**^*P* < 0.01 versus Model group; One-way ANOVA was used to calculate the p-values. The average swimming distance and escape latency of mice in the Morris Water Maze experiment were repeated measurements, both of which were in line with the spherical test, and a repeated-measures ANOVA was applied

To assess spatial learning and memory, MWM test was performed after 3 months of treatment. In different quadrants, mice in SZL groups tended to be closer to the platform and have a shorter swimming distance (Fig. 4A, B). Furthermore, the average swimming distance and the mean latency avoidance period gradually shortened in all groups with the progression of test days in the directional navigation experiment. There was a significant effect over time for SZL treatment. Especially, the average swimming distance and the average escape latency of mice in SZL groups was shorter on days 4 to 5 (*P* < 0.05; Fig. 4C, D). At last, the time spent and times of crossing platform in the target quadrant was longer in SZL groups compared with model group in the space exploration experiment. (*P* < 0.01, *P* < 0.05; Fig. 4E, F). These data indicate that SZL improves cognitive function and memory in APP/PS1 double transgenic mice.

### SZL modulate the pathology and protein expression of Aβ_42_

**Fig. 5:**
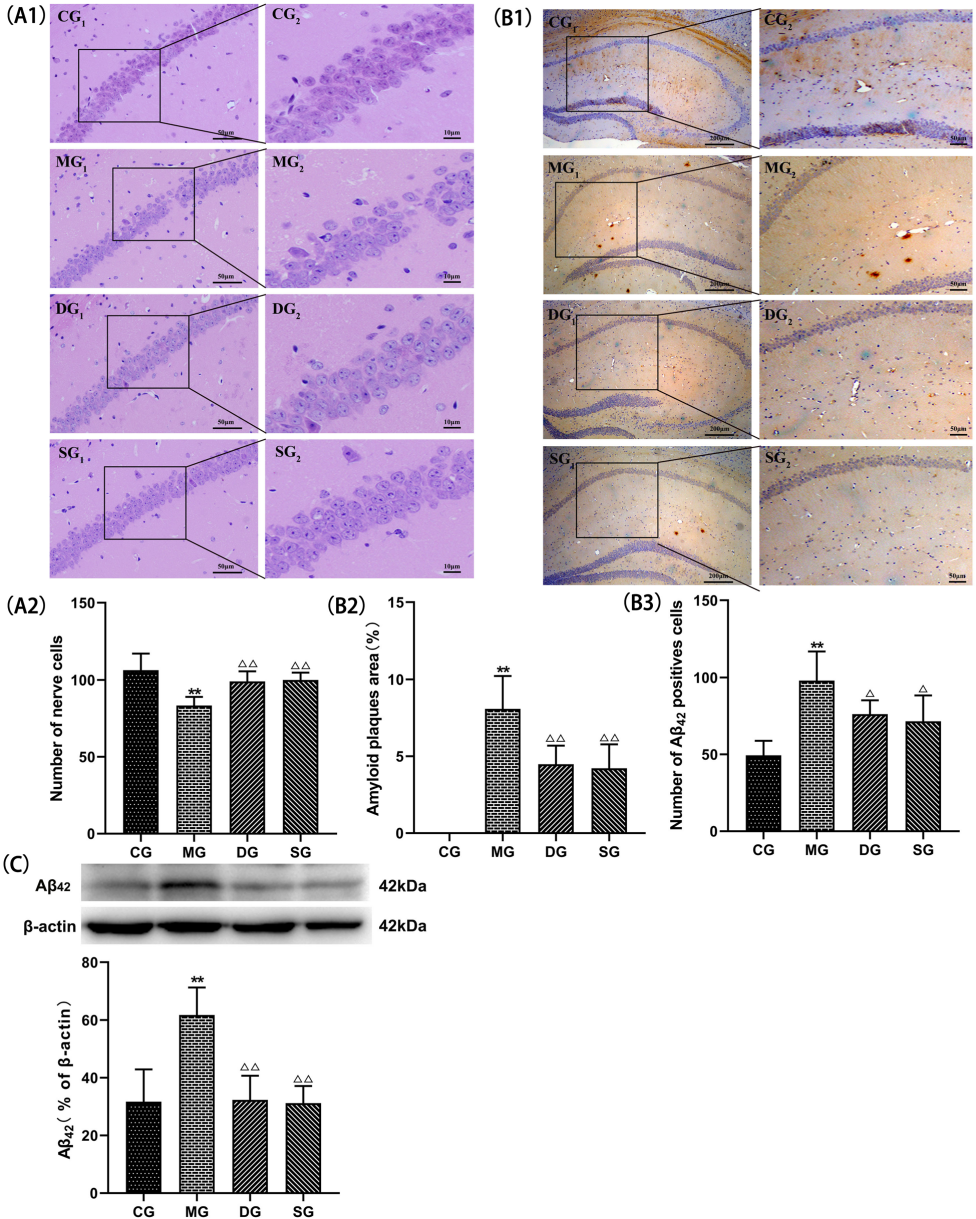
Effect of SZL on the main structure and the expression of Aβ_42_ in APP/PS1 mice. Statistical analysis of positive cells of HE (**A1**, **A2**; scale bar = 50 μm; *n* = 6). Statistical analysis of amyloid plaques area (**B1**–**B3**; scale bar = 200 μm; *n* = 3). Statistical analysis of protein expressions of Aβ_42_ (**C**). All data are presented as means ± SEM.CG: control group, MG: model group, DG: donepezil group, SG: SZL group. *P < 0.05, **P < 0.01 versus Control group, ^**△**^*P* < 0.05, ^**△△**^*P* < 0.01 versus Model group; One-way ANOVA was used to calculate the p-values

We used HE staining to measure neurons in the hippocampus of mice. The number of neurons in the model group decreased and the structure was loose or even non-existent compared with control group. The injured neurons in SZL group were repaired to some extent, and the number of cells was increased and the cells were closely arranged compared with model group (*P* < 0.01; Fig. 5A1, A2). To observe the effects of SZL on the pathological changes of APP/PS1 double transgenic mice, the expression of Aβ_42_ by Immunohistochemical staining and Western blotting analysis. There were fewer amyloid plaques, positive neuron (*P* < 0.05 or *P* < 0.01; Fig. 5B1–B3) and lower protein expression of Aβ_42_ in the SZL group (*P* < 0.05; Fig. 5C).

### SZL improved the ultrastructural changes related to glucose metabolism

**Fig. 6:**
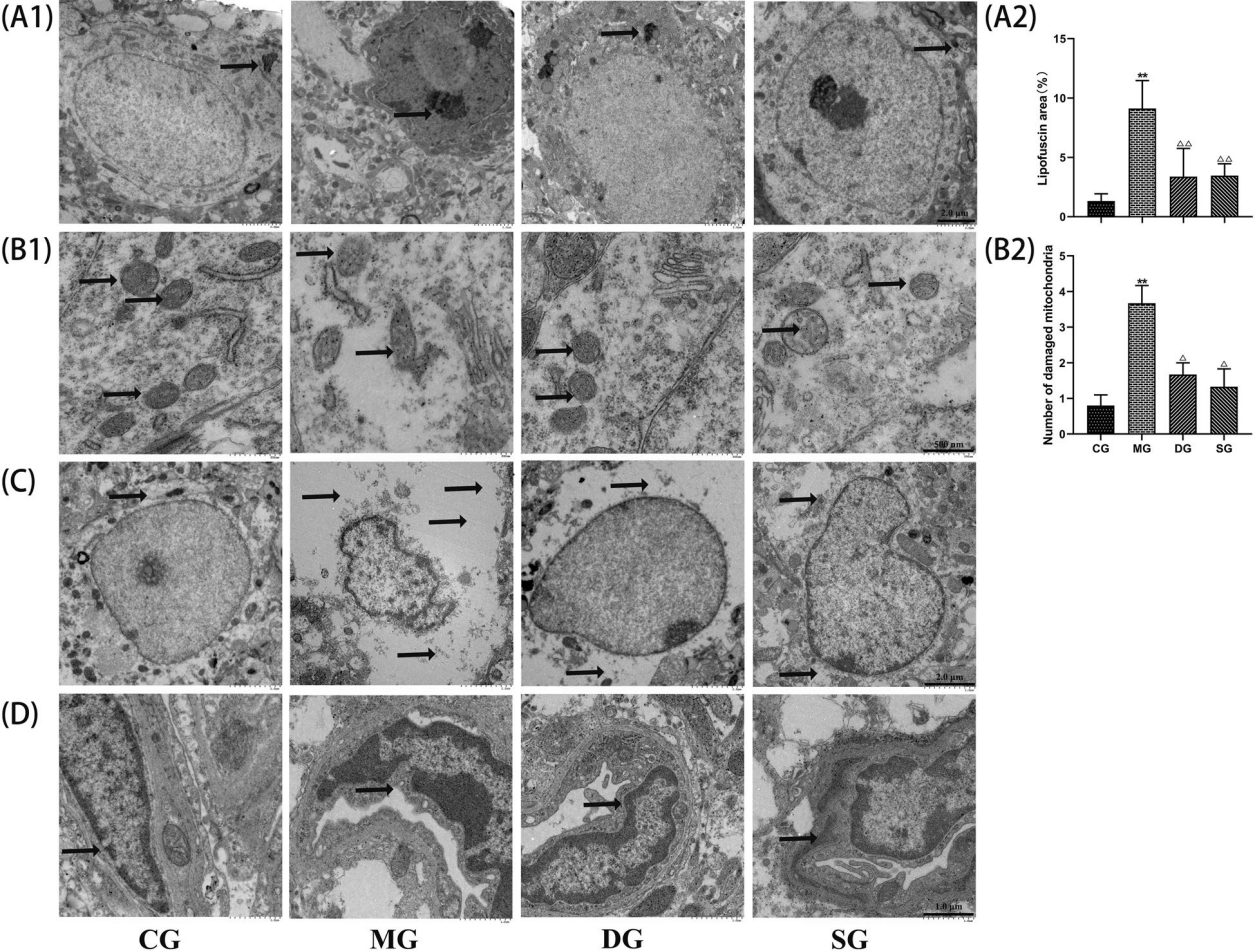
Effect of SZL on hippocampal ultrastructural in APP/PS1 mice. **A** Ultrastructure of neurons (× 1.5 k, scale = 2.0 μm, *n* = 3), **B** Ultrastructure of neuronal organelles (× 8 k, scale = 500 nm, *n* = 3), **C** Ultrastructure of astrocytes (× 2 k, scale = 2.0 μm, *n* = 3), **D** Ultrastructure of microvascular (× 4 k, scale = 1.0 μm, *n* = 3). Statistical analysis of Lipofuscin area (**A1**) and Number of damaged mitochondria (**B1**). All data are presented as means ± SEM.CG: control group, MG: model group, DG: donepezil group, SG: SZL group. *P < 0.05, **P < 0.01 versus Control group, ^**△**^*P* < 0.05, ^**△△**^*P* < 0.01 versus Model group; One-way ANOVA was used to calculate the p-values

To further determine the effect of SZL on morphological changes of neurons damage, we assessed the cell morphology of hippocampal CA1 neurons in these mice. In the control group, the structure of hippocampal neurons was intact, with normal nuclear morphology, abundant intracytoplasmic organelles, intact mitochondria, and rough endoplasmic reticulum and ribosomes (Arrow in Fig. 6B1, B2). In the model group, the neurons exhibited pyknosis, nuclear chromatin concentration and edge aggregation, polyribosome depolymerization, and swollen mitochondria, as well as some mitochondria rupture, more lipofuscin (Arrow in Fig. 6A1, A2), the whole cell showed degenerative changes. The structure and morphology of hippocampal neurons in the SZL group were roughly normal, mitochondria were swollen, and Golgi, rough endoplasmic reticulum, and polyribosome were basically intact (Arrow in Fig. 6A1, B1).

In the control group, the astrocytes were clearly defined, with large nuclei and large flat round cell bodies, abundant cytoplasm. In the model group, the hippocampal astrocytes showed seriously pyknosis and edema (Arrow in Fig. 6C), with vacuoles in the cytoplasm, nuclear pyknosis, and a fuzzy nuclear membrane structure. Hippocampal astrocytes in the SZL group showed slight edema, with more euchromatin that was evenly dispersed, and heterochromatin blocks distributed along the nuclear membrane (Arrow in Fig. 6C).

In the control group, the endothelial cells of the hippocampus micrangium were intact, the basal membrane of the vessels was intact, the structural hierarchy was clear, and the surrounding tissues were closely arranged. In the model group, the endothelial cells (Arrow in Fig. 6D) of the hippocampus micrangium were distorted and deformed, with severe edema in the official lumen, a thickened basement membrane, blurred boundary and structure, and loose arrangement of surrounding tissues (Arrow in Fig. 6D). In the SZL group, the basal membrane of the tube was complete, the contour was fuzzy and irregular, and the structure was clear (Arrow in Fig. 6D).

### SZL regulates the insulin signal transduction pathway

#### SZL improve the expression of InR, p-InR, IRS2, and p-IRS2

**Fig. 7:**
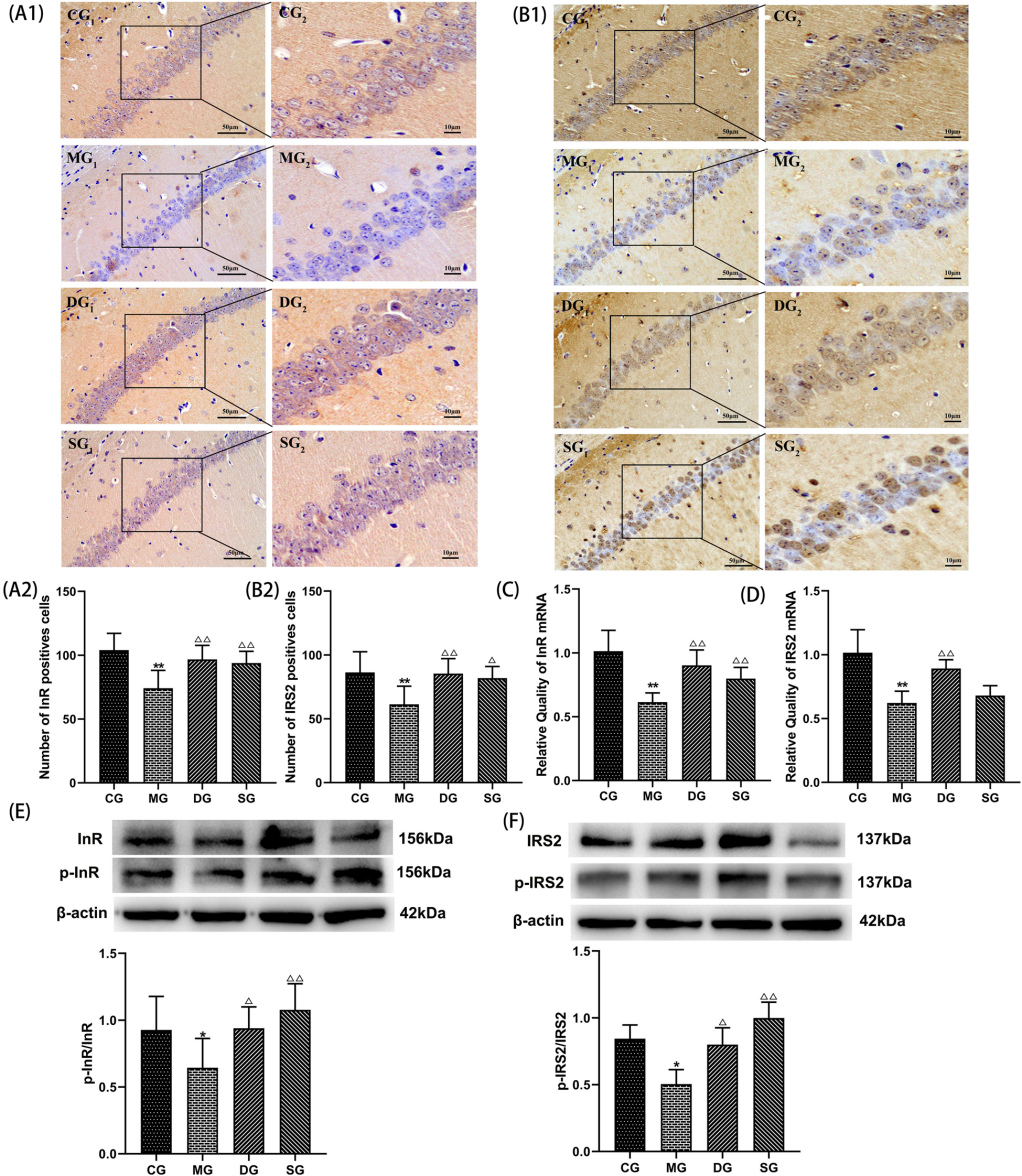
Effect of SZL on the expression of InR, p-InR, IRS2, p-IRS2 in hippocampus in APP/PS1 mice. Statistical analysis of positive cells of InR (**A1**, **A2**), IRS2 (**B1**, **B2**) (*n* = 6). Statistical analysis of mRNA expression of InR (**C**), IRS2 (**D**) (*n* = 3). Statistical analysis of protein expressions of p-InR/ InR(E). p-IRS2/ IRS2 (**F**) (*n* = 3).CG: control group, MG: model group, DG: donepezil group, SG: SZL group. All data are presented as means ± SEM.*P < 0.05, **P < 0.01 versus Control group, ^**△**^*P* < 0.05, ^**△△**^*P* < 0.01 versus Model group; One-way ANOVA was used to calculate the p-values

To elucidate the mechanisms underlying the neuroprotective effect of SZL, the expression of InR, p-InR, IRS2, and p-IRS2 by immunohistochemical, RT-PCR and western blotting analysis. Immunohistochemical results showed that InR was mainly expressed in the cell membrane of neurons. There were significantly fewer InR, IRS2-positive cells and lower expression of InR, IRS2 mRNA in the model group than in the control group (*P* < 0.01; Fig. 7A–D). In turn, there were significantly more InR, IRS2-positive cells and higher expression of InR, IRS2 mRNA in the donepezil and SZL groups than in the model group (*P* < 0.01 or *P* < 0.05; Fig. 7A–D). In addition, SZL augmented phosphorylation of InR, IRS2 compared with the model group (*P* < 0.01; Fig. 7E, F).

#### SZL regulates the PI3K/Akt/GSK3β pathways

**Fig. 8:**
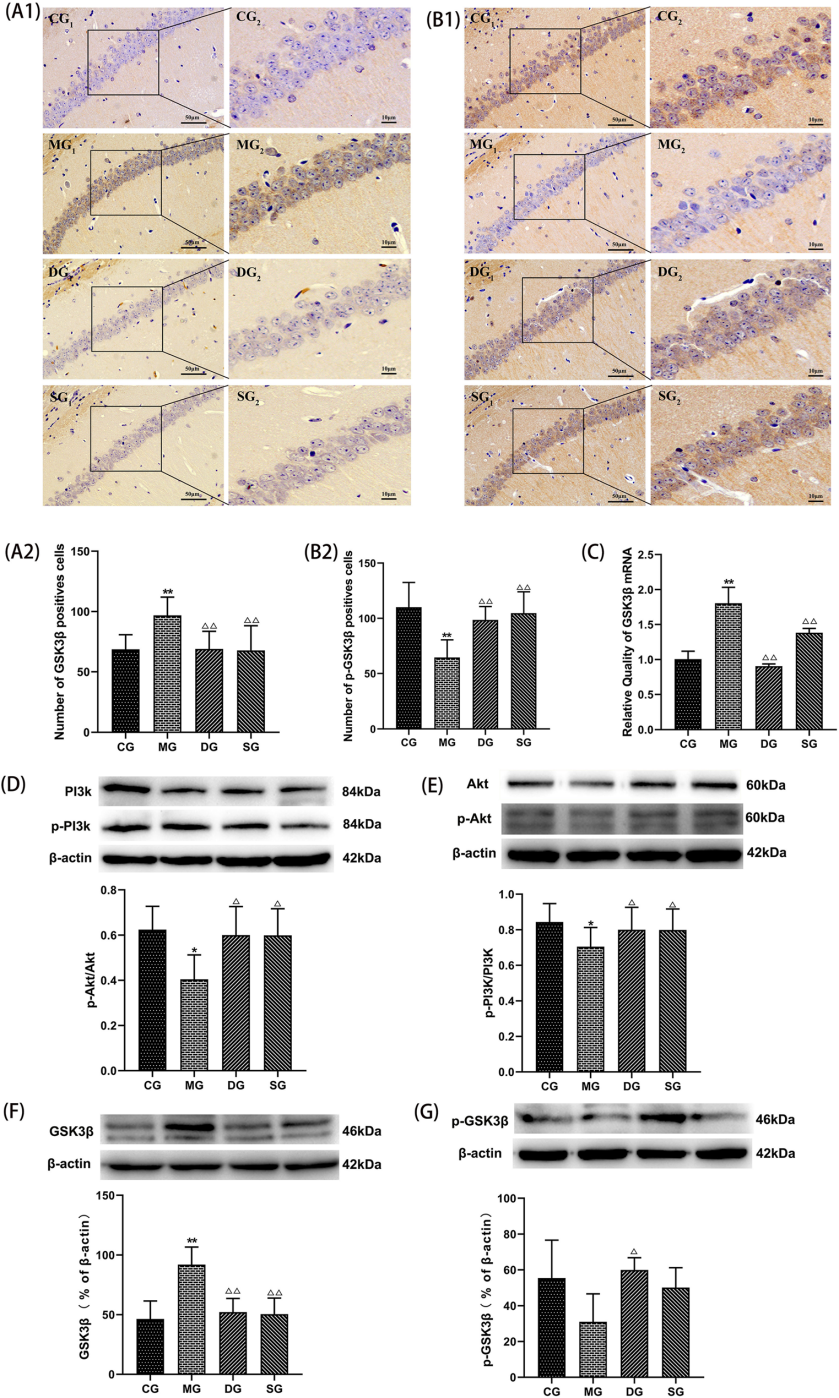
The effect of SZL on PI3K/Akt/GSK3β pathway. Statistical analysis of positive cells of GSK3β (**A1**, **A2**), p-GSK3β (B1, B2) (*n* = 6). Statistical analysis of mRNA expression of GSK3β (**C**) (*n* = 3). Statistical analysis of protein expressions of p-PI3K/PI3K (**D**), p-Akt/Akt (**E**), GSK3β (**F**), p-GSK3β (**G**) (*n* = 3).CG: control group, MG: model group, DG: donepezil group, SG: SZL group. All data are presented as means ± SEM. *P < 0.05, **P < 0.01 versus Control group, ^**△**^*P* < 0.05, ^**△△**^*P* < 0.01 versus Model group; One-way ANOVA was used to calculate the p-values

To elucidate the mechanisms underlying the neuroprotective effect of SZL, the expression of key proteins related with PI3K/Akt/GSK3β signaling pathways by immunohistochemical, RT-PCR and western blotting analysis. SZL augmented phosphorylation of PI3K, Akt compared with the model group (*P* < 0.05; Fig. 8 D, E). GSK3β was mainly expressed in the membrane of neuronal cells. Compared with model group, the number of GSK3β positive cells in SZL group was significantly increased (*P* < 0.01; Fig. 8A1, A2). Compared with the control group, the expression of GSK3β protein and GSK3β mRNA in model group were increased (*P* < 0.01; Fig. 8B1, B2). Compared with model group, the expression of GSK3β protein and GSK3β mRNA in SZL groups were decreased (*P* < 0.01; Fig. 8C, F). On the contrary, p-GSK3β (Ser 9) showed an opposite trend change (Fig. 8G).

### SZL enhanced the expression of glucose transporter-related proteins and glycolysis-related genes

**Fig. 9:**
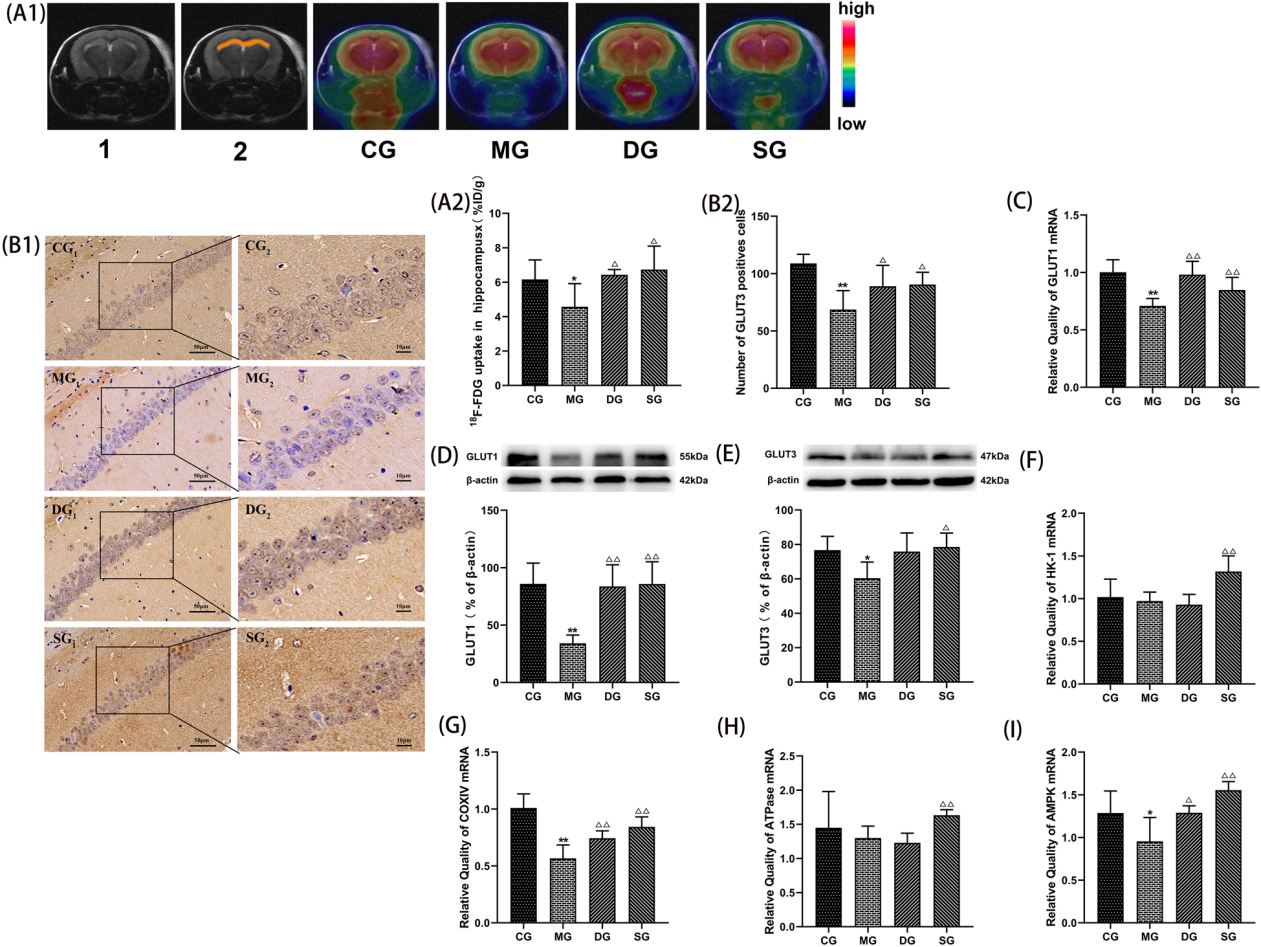
Effect of SZL on glucose transporter-related proteins and glycolysis-related genes. Statistical analysis of hippocampal glucose uptake (**A1**, **A2**): 1.Standard mouse brain template localization.2. Hippocampal positioning (*n* = 3). Statistical analysis of positive cells of GLUT3 (**B1**, **B2**), Statistical analysis of protein expressions of GLUT1 (**D**), GLUT3 (**E**) (*n* = 6). Statistical analysis of mRNA expression of GLUT1 (**C**), HK1 (**F**), COXIV (**G**), ATPase (**H**), AMPK (**I**) (*n* = 3).CG: control group, MG: model group, DG: donepezil group, SG: SZL group. All data are presented as means ± SEM.*P < 0.05, **P < 0.01 versus Control group, ^**△**^*P* < 0.05, ^**△△**^*P* < 0.01 versus Model group; One-way ANOVA was used to calculate the p-values

The change of glucose uptake from high to low revealed by the micro-PET images is shown in Fig. 9. The hippocampal color of model group was yellow, and the glucose intake of grapes was reduced compared with that of the control group, shown in red (*P* < 0.05; Fig. 9A1, A2). Compared with the model group, the hippocampal glucose uptake was higher SZL groups was higher, shown in red (*P* < 0.05; Fig. 9A1, A2). Immunohistochemical results showed that glucose transporter GLUT3 was mainly expressed in the membrane of neuronal cells. SZL significantly improve the expression GLUT3-positive cells number and the expression of GLUT3 protein (*P* < 0.05; Fig. 9B1, B2). SZL dramatically promote the elevated levels of GLUT1 mRNA and protein expression (*P* < 0.01; Fig. 9C, D). In the present study, SZL markedly increased HK1, COXIV, ATPase, and AMPK mRNA expression. (*P* < 0.01; Fig. 9F–I). The data suggest that SZL could markedly improve brain glucose and uptake. Collectively, these results suggested that SZL could play an anti-amnesic role in APP/PS1 mice by its likely ability to regulate the Insulin signaling pathway InR/PI3K/Akt/GSK3β.

### SZL-containing serum regulate the PI3K/Akt/GSK3β pathways

**Fig. 10:**
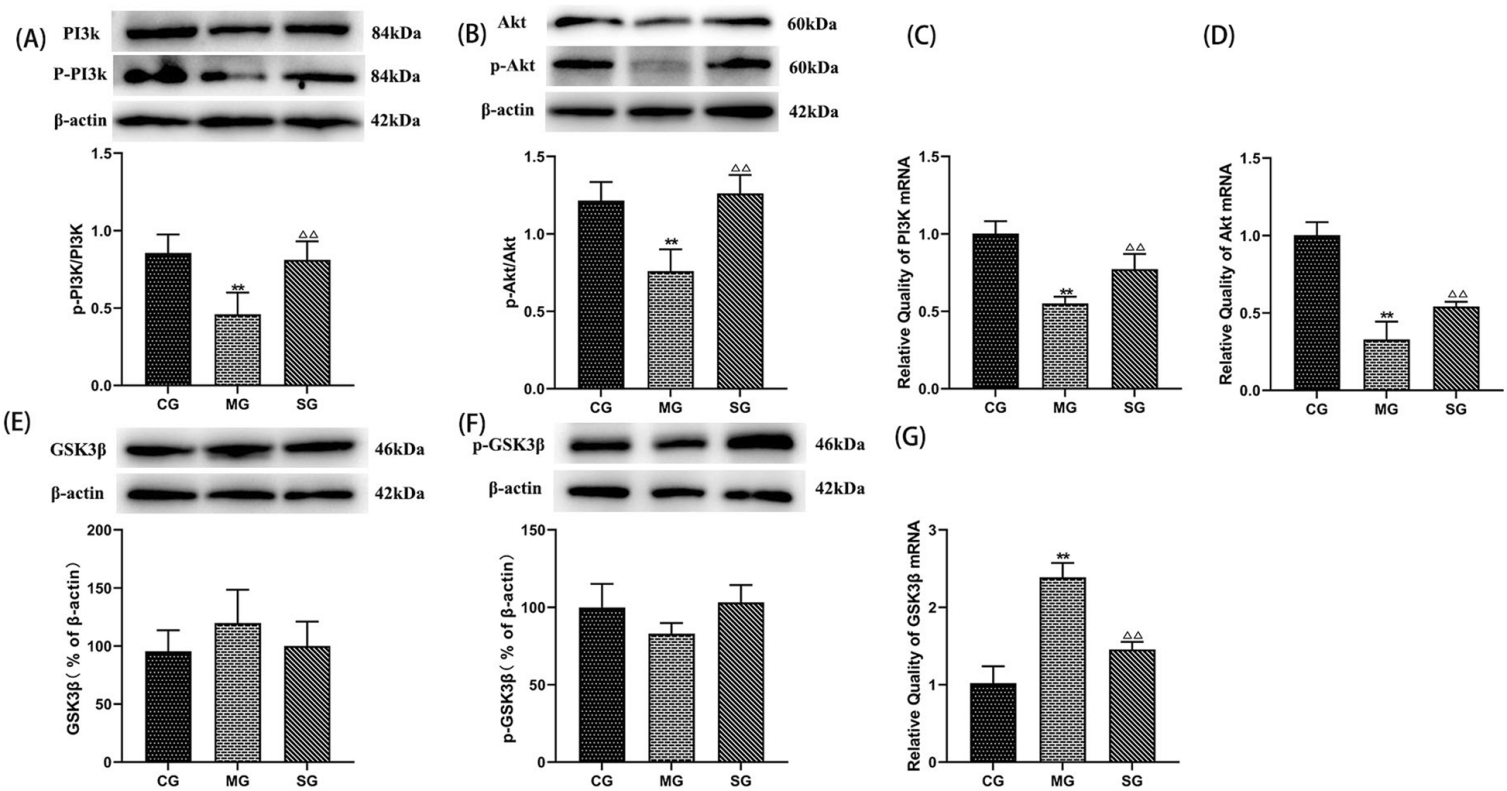
Effect of SZL-containing serum on the expression of insulin signal transduction pathway. Statistical analysis of protein expressions of p-PI3K/PI3K (**A**), p-Akt/Akt (**B**), GSK3β (**E**), p-GSK3β (**F**). Statistical analysis of mRNA of PI3K (**C**), Akt (**D**), GSK3β (**G**). CG: control group. All data are presented as means ± SEM (*n* = 6). *P < 0.05, **P < 0.01 versus Control group, ^**△**^*P* < 0.05, ^**△△**^*P* < 0.01 versus Model group; One-way ANOVA was used to calculate the p-values

In cell study, the activation of the PI3K/Akt pathway after SZL-containing serum treatment were further evaluated to better understand how SZL exerts protective effect against AD. The specific cell modeling method of this study was shown in Additional file 1. As depicted in Fig. 10 Aβ_42_ lead to the lower protein expression levels of p-PI3K, p-Akt and gene expression of PI3K, Akt, while SZL-containing serum improved this effect obviously (*P* < 0.01; Fig. 10A–D). Futhermore, SZL-containing serum down-regulated the expression of protein and gene of GSK3β, and increase the expression of p-GSK3β (*P* < 0.01; Fig. 10E–G).

### SZL-containing serum regulate the glucose transporter

**Fig. 11:**
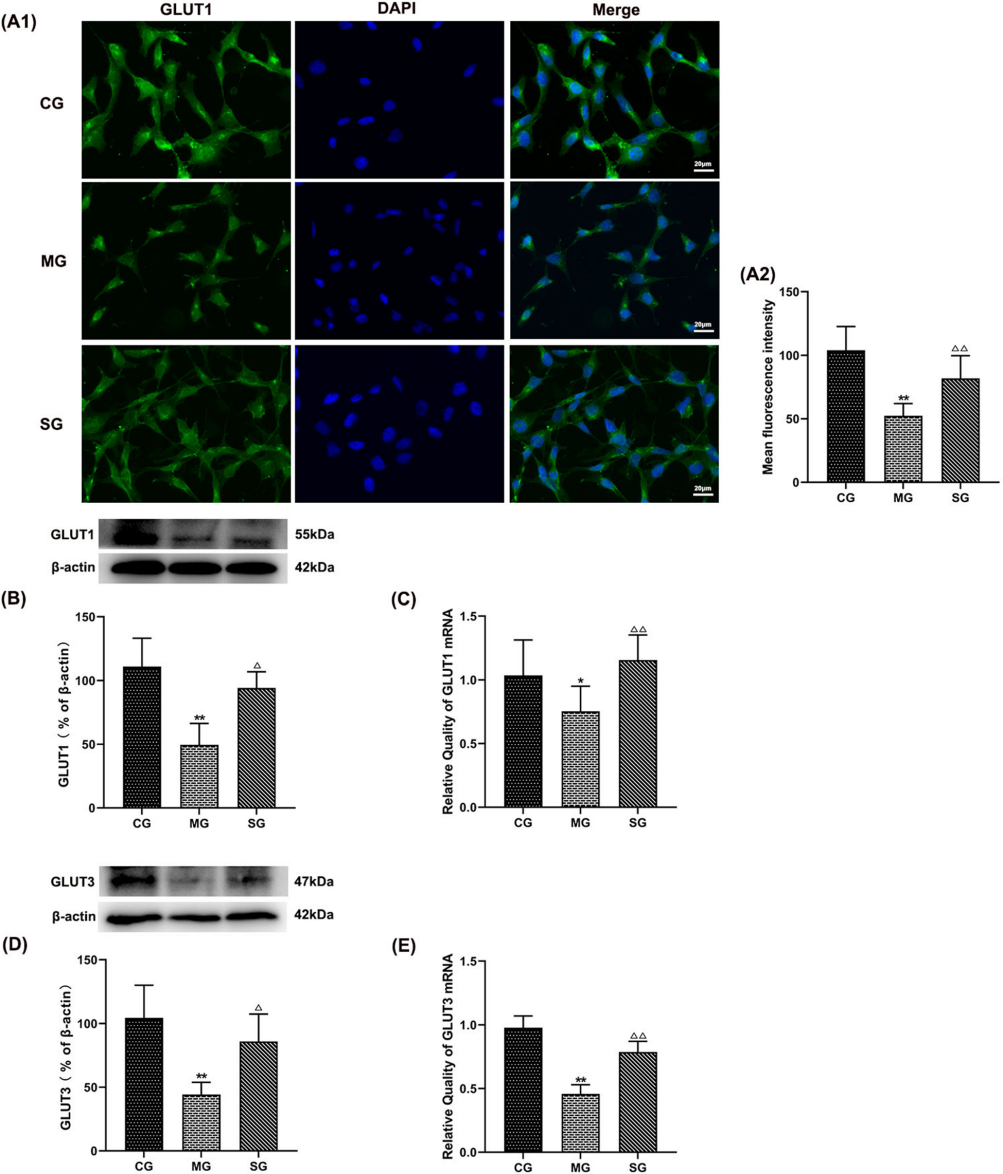
Effect of SZL-containing serum on the expression of dysfunction of CLUTs. Statistical analysis of fluorescence intensity of GLUT1 (**A1**, **A2**). Statistical analysis of protein expressions of GLUT1 (**B**), GLUT3 (**D**). Statistical analysis of mRNA expression of GLUT1 (**C**), GLUT3 (**E**).CG: control group. All data are presented as means ± SEM (*n* = 6). *P < 0.05, **P < 0.01 versus Control group, ^**△**^*P* < 0.05, ^**△△**^*P* < 0.01 versus Model group, ^▼^*P* < 0.05, ^▼▼^*P* < 0.01 versus SZL-containing serum group; One-way ANOVA was used to calculate the p-values

As depicted in Fig. 11, Aβ_42_ lead to the lower protein and mRNA expression levels of GLUT1, GLUT3, while SZL-containing serum improved this effect obviously (*P* < 0.05 or *P* < 0.01; Fig. 11C–F). In addition, fluorescence microscopy was applied to the observation of GLUT1 production. Results exhibited that SZL-containing serum reduced GLUT1 fluorescence intensity dramatically (*P* < 0.01; Fig. 11A, B), which corresponded to the above results.

### SZL-containing serum and the use of PI3K and/or GSK3β inhibitor.

**Fig. 12:**
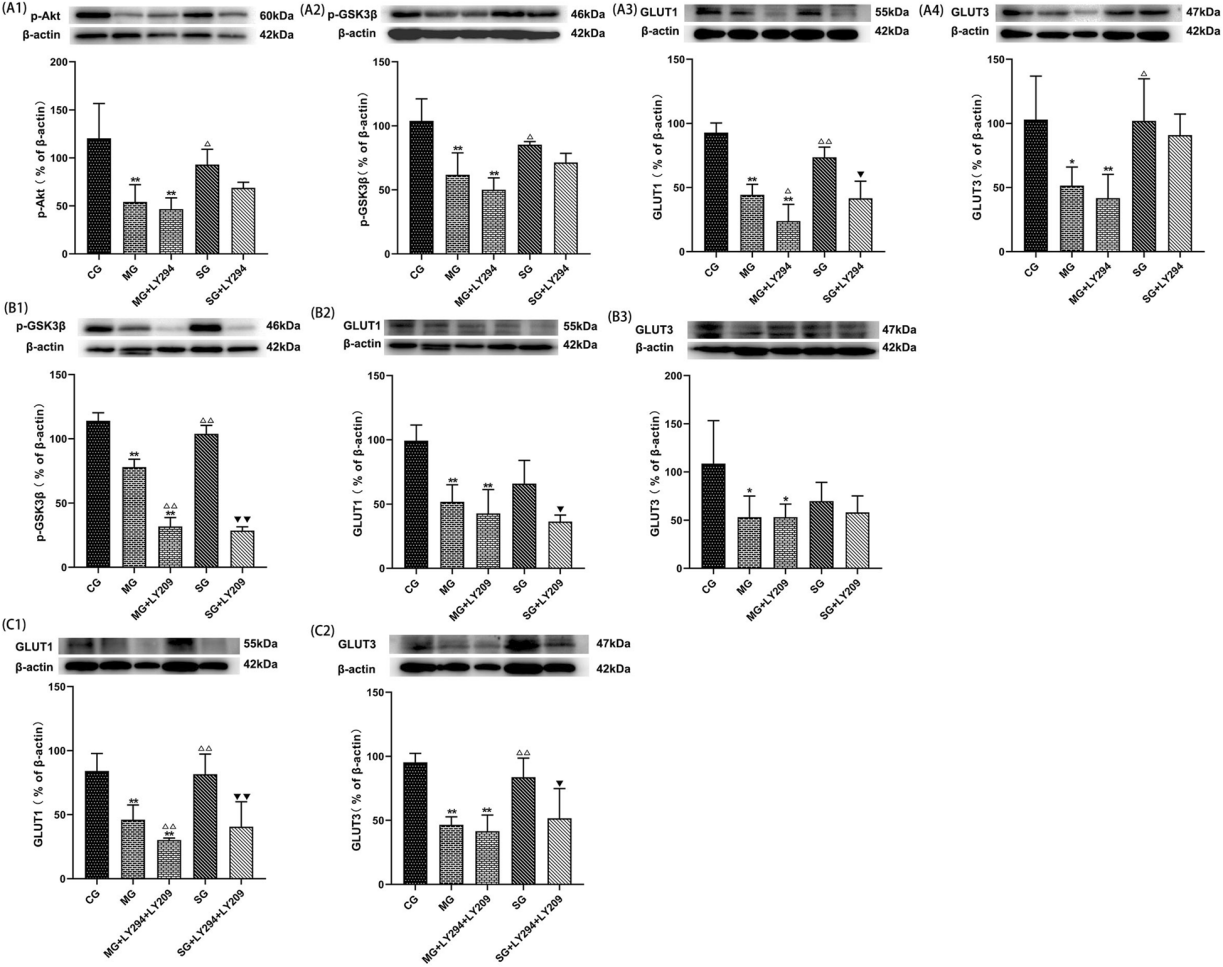
Effects of SZL-containing serum with PI3K and/or GSK3β inhibitor. Statistical analysis of protein expressions of p-Akt (**A1**), p-GSK3β (**A2**), GLUT1 (**A3**), GLUT3 (**A4**) with PI3K inhibitor. Statistical analysis of protein expressions of p-GSK3β (**B1**), GLUT1 (**B2**), GLUT3 (**B3**) with GSK3β inhibitor. Statistical analysis of protein expressions of GLUT1 (**C1**), GLUT3 (**C2**) with PI3K and GSK3β inhibitor. CG: control group. All data are presented as means ± SEM (*n* = 6). *P < 0.05, **P < 0.01 versus Control group, ^**△**^*P* < 0.05, ^**△△**^*P* < 0.01 versus Model group; ^▼^*P* < 0.05, ^▼▼^*P* < 0.01 versus SZL-containing serum group; One-way ANOVA was used to calculate the p-values

PI3K/Akt/GSK3β pathway is a classical pathway regulating glucose metabolism. In this study, the use antagonists of PI3K and GSK3β were further evaluated to better understand how SZL exerts protective effect against cerebral glucose metabolism. The results showed that the two inhibitors, either alone or in combination, could weaken the repair effect of SZL-containing serum to different degrees (*P* < 0.05, or *P* < 0.01; Fig. 12). These results suggest that SZL may play a glucose metabolism role by activating the PI3K/Akt/GSK pathway.

## Discussion

The active component of the Chinese medicine compound SZL has neuroprotective effects [9, 12]. In one study of hypoglycemic effects, a neutral polysaccharide extracted from Codonopsis pilosula residue was found to ameliorate lipid metabolism and increase glycolytic enzyme activity [14]. Glycyrrhetinic acid protects H9c2 cells from oxygen glucose deprivation-induced injury, and the potential underlying mechanism might be related to the PI3K/Akt signaling pathway [15]. The rhizome of Polygala tenuifolia WILLD has been used in Traditional Chinese Medicine to treat inflammation, dementia, amnesia, neurasthenia, and cancer [16]. A novel polysaccharide from Acorus tatarinowii has been found to exert anti-neuroinflammatory and neuroprotective effects through modulation of the PI3K/Akt signaling pathway [17]. As we all know, it is hard to tell which compound exerts primary effects in herb formula due to its feature of multi-ingredients [18]. LC–MS/MS was used to identify the active components of SZL and SZL-containing serum. Interestingly, SZL peak values were inconsistent with the peak values of SZL drug-containing serum. In line with the above studies, we found that SZL not only improved the learning and performance of AD model mice, but also that the major components of related drugs influenced glucose metabolism and the PI3K/Akt signaling pathway.

One of the typical pathological features of AD is the formation of Aβ plaques in the brain caused by the aggregation of Aβ peptides. The Aβ hypothesis is the classical hypothesis of AD pathogenesis [19]. APP/PS1 transgenic mice simulate the pathological characteristics of AD, and this universally recognized transgenic animal model has been used to study AD etiology and prevention strategies [20]. In this study, SZL can repair damaged neurons and reduce the accumulation of Aβ. These preliminarily experimental results indicate that SZL can improve the learning and memory ability of AD model mice.

Recent evidence indicates an intimate connection between brain insulin resistance and the formation of Aβ oligomers. Relevant studies show that the treatment of primary hippocampal neurons by soluble Aβ oligomers results in a profound loss of insulin receptors from the neuronal surface [21]. In addition, Aβ oligomers inhibit insulin signaling by phosphorylating IRS at inhibitory serine residues [22]. Insulin resistance facilitates the formation of Aβ. Increasing evidence has shown that insulin is a key factor in the control of blood glucose levels, which promotes glucose uptake in peripheral tissues by activating the PI3K/Akt pathway. The InR/PI3K/Akt signaling pathway plays an important role in neuronal integrity and synaptic formation and maintenance [23, 24]. In the insulin sensitive state insulin binds to the receptor and activates the insulin receptor tyrosine kinase that initiates a cascade of phosphorylation events at the InR/PI3K/Akt pathways [2]. The PI3K/Akt pathway regulates various cellular functions through downstream factors. GSK3β is one of the most important phosphorylated tau kinases. Ser 9 is a GSK3β inhibition site, while Tyr 216 is a GSK3β activated site. Thus, phosphorylation of GSK3β at Ser 9 leads to inhibition of GSK3β, while dephosphorylation at this site leads to activated GSK3β. Insulin-activated PI3K/Akt signaling negatively regulates GSK3β by increasing or decreasing Ser 9 phosphorylation. Insulin has been shown to effectively increase the phosphorylation of GSK3β by Ser 9, thereby inactivating GSK3β by upregulation [25, 26]. Beyond that in a state of insulin resistance, GSK-3β is restrained by phosphorylation at Ser 9 residue and affect glucose transporter expression [2].

GLUT1 mediates the transport of glucose from the blood to the brain. GLUT1 is located on the endothelial cells of the blood brain barrier. Previous work has shown that the decreased expression of GLUT1 in the brain of APP/PS1 mice is closely related to the deposition of Aβ plaques [27]. GLUT3 is located in neurons, and decreased GLUT3 levels lead to decreased glucose uptake and metabolism. A previous study found that the decreased expression levels of GLUT1 and GLUT3 in the cerebral cortex of patients with AD impairs glucose uptake in the brain, thus leading to the hyperphosphorylation of tau protein [28]. Therefore, the abnormality of the glucose transporter plays an important role in the pathogenesis of AD. The present study revealed that SZL improved the expression of the insulin signal transduction pathway and glucose transporter to varying degrees [29]. These preliminarily results indicate that the neuroprotective effect of SZL in AD model mice is related to brain glucose metabolism, and its mechanism may be related to the insulin signal transduction pathway.

Astrocytes are the most abundant glial cells in the central nervous system and are essential for maintaining a healthy brain structure and function [30]. Astrocytes are surrounded by capillaries, and capillaries and interact with endothelial cells through their neurovascular terminus. Beyond that, astrocytes connect synapses and capillaries, which are the cellular connections between neuronal activity and blood vessels. Astrocytes also participate in maintaining the normal function of blood brain barrier through GLUTs [31, 32]. In our study, we observed the ultrastructure of hippocampus of different groups of mice using electron microscopy, and showed that SZL could improve the ultrastructure of neurons, mitochondria, microvessels, and astrocytes of APP/PS1 double transgenic mice to a certain extent. We can speculate that the mechanism of amelioration of AD may be related to the repair of mitochondria, astrocytes, and microvessels involved in brain glucose metabolism.

Hexokinase (HKs) catalyzes the first step of glucose metabolism by phosphorylating glucose to glucose 6-phosphate [33]. Among the HKs, HK1 plays a major role in this process. HK1 promotes glucose phosphorylation, which can effectively prevent glucose leakage from cells; this can guarantee glucose energy metabolism in cells and thus play a neuroprotective role [34]. HK1 glucose phosphate is converted to glucose-6-phosphate in the initial step of glycolysis, whereby the pyruvate produced can be transported to the mitochondria and then converted to acetyl-CoA by pyruvate dehydrogenase, which drives the TCA cycle. COX is the terminal complex of eukaryotic oxidative phosphorylation in mitochondria, and the regulatory site of the mitochondrial oxidative phosphorylation system [35]. COX deficiency is a widely recognized cause of mitochondrial diseases in humans and can affect ATP production. In addition, COX functional changes play an important role in the occurrence and development of AD [36]. ATP synthetase is a key enzyme in the synthesis of ATP, provides energy for organisms, and participates in many intercellular signal transmission processes [37]. The present study found that the expression of HK1, a key enzyme involved in glucose metabolism (COXIV) and ATP synthesis (ATPase and AMPK) in the brain of mice in the model group decreased by different degrees, which was improved by SZL.

Human neuroblastoma SH-SY5Y is a dopaminergic neuronal cell line which has been used as an in vitro model for in vitro neuroscience studies since its first discovery [38]. In the in vitro cell experiments, Aβ_42_-damaged SH-SY5Y cells were used to construct an in vitro model of AD, which corresponded to the APP/PS1 double transgenic mouse model used in the animal experiments. The up-regulation of GLUT1 and GLUT3 proteins in the SZL-containing serum group could be reversed to different degrees. Antagonists of PI3K and GSK3β were added in the cell experiments to further confirm the regulatory role of the insulin signal transduction pathway in the improvement of brain glucose metabolism by SZL.

## Conclusions

From above results, we can conclude that the improvement of glucose uptake and transport in the brain may be the pathway by which SZL exerts its neuroprotective effect, and the potential molecular mechanism may be related to the normalization of the insulin signal transduction pathway InR/PI3K/Akt in early AD. These underlying mechanisms highlighted that SZL owned the advantage of promising memory protection activities, which facilitated the development of a novel agent for the treatment of AD.

## Supplementary Information

**Additional file 1.** The specific modeling method and model identification.

**Additional file 2.** Identification of components of SZL into blood.

## Data Availability

The data used and/or investigated during the present study are available from the corresponding author upon reasonable request.

## Abbreviations

AD: Alzheimer’s disease
SZL: Shen-Zhi-Ling oral liquid
Aβ: Beta-amyloid
InR/PI3K/Akt: Insulin-like receptor/phosphoinositide 3-kinase/protein kinase B
micro-PET: Micro-positron emission tomography
PBS: Preheated phosphate-buffered saline
RT-qPCR: Reverse transcription quantitative real-time polymerase chain reaction
RT-PCR: Reverse transcription polymerase chain reaction
GSK3β: Glycogen Synthase Kinase 3β
InR: Insulin receptor
IRS: Insulin receptor substrate
PI3K: Phosphatidylinositol -3- kinase
