# Correction to: Shen-Zhi-Ling oral liquid ameliorates cerebral glucose metabolism disorder in early AD via insulin signal transduction pathway in vivo and in vitro

**DOI:** 10.1186/s13020-021-00551-x

**Published:** 2021-12-31

**Authors:** Gaofeng Qin, Yunfang Dong, Zhenhong Liu, Zhuoyan Gong, Chenyan Gao, Mingcui Zheng, Meijing Tian, Yannan He, Liqun Zhong, Pengwen Wang

**Affiliations:** 1 Key Laboratory of Chinese Internal Medicine of Ministry of Education and Beijing, Dongzhimen Hospital, Beijing University of Chinese Medicine (BUCM), Haiyuncang No. 5 in Dongcheng District, Beijing, China; 2 Binzhou Medical University Hospital, Shandong, China; 3 Institute for Brain Disorders, Beijing University of Chinese Medicine (BUCM), Beijing, China; 4 Beijing Prominion Publishing Co. Ltd, Beijing, China

## Correction to: Chinese Medicine (2021) 16: 128 https://doi.org/10.1186/s13020-021-00540-0

Following publication of the original article [1], the authors identified an error in the column DAPI of Fig. 11 (A1). The correct Fig. 11 (Fig. 11) is given in this erratum.

**Fig. 11:**
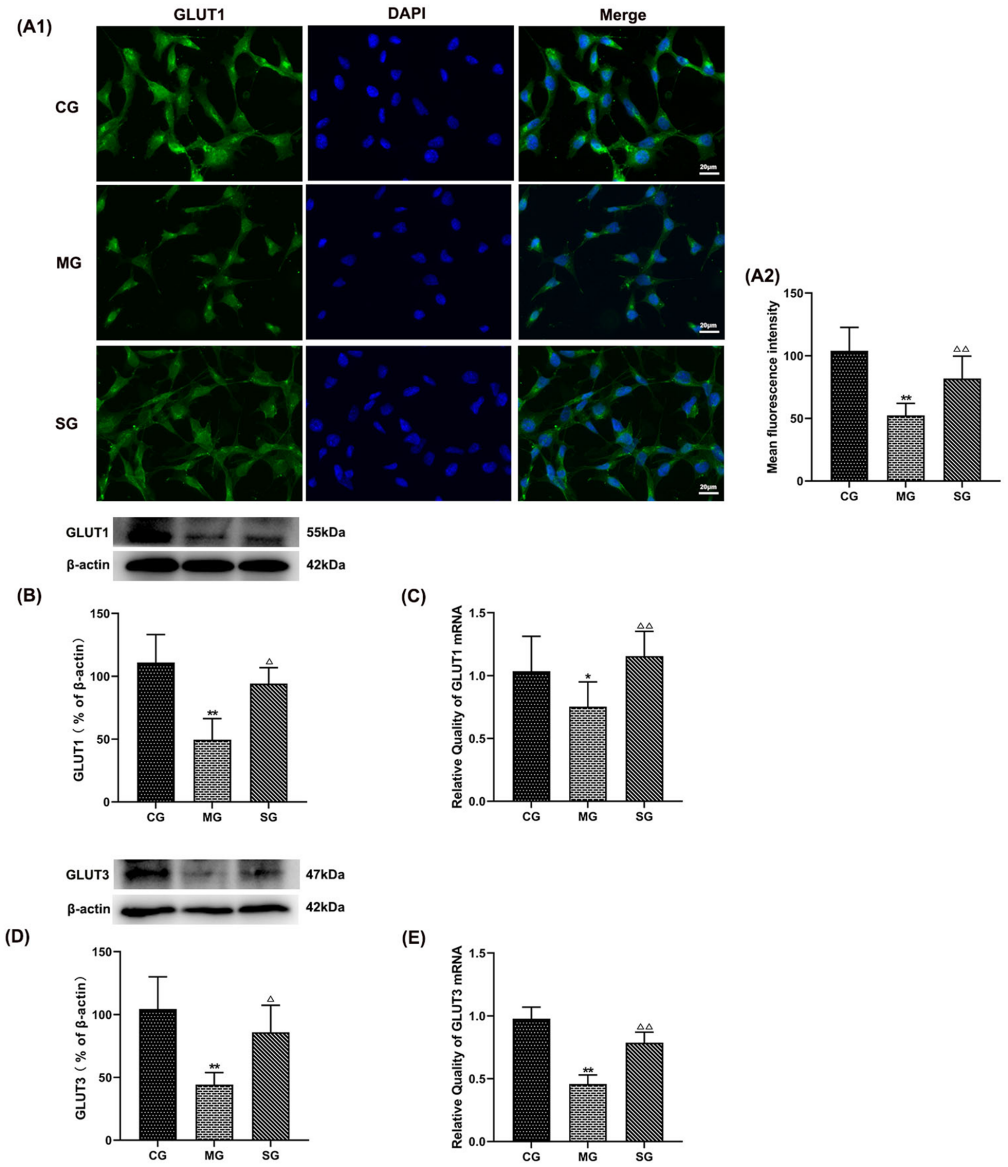
Effect of SZL-containing serum on the expression of dysfunction of CLUTs. Statistical analysis of fluorescence intensity of GLUT1 (**A1**, **A2**). Statistical analysis of protein expressions of GLUT1 (**B**), GLUT3 (**D**). Statistical analysis of mRNA expression of GLUT1 (**C**), GLUT3 (**E**).CG: control group.All data are presented as means ± SEM (n = 6). *P  < 0.05, **P < 0.01 versus Control group, ^△^P < 0.05, △△P < 0.01 versus Model group, ^▼^ P< 0.05, ^▼▼^P < 0.01 versus SZL-containing serum group; one-way ANOVA was used to calculate the p-values

The original article has been corrected.
