# Supplementary material for: Shen-Zhi-Ling oral liquid ameliorates cerebral glucose metabolism disorder in early AD via insulin signal transduction pathway in vivo and in vitro
Source: Chin Med. 2021 Dec 2;16:128. doi: 10.1186/s13020-021-00540-0 (PMC8638512; doi:10.1186/s13020-021-00540-0)
Supplement: Supplementary file 1 — Additional file 1. The specific modeling method and model identification. [file 13020_2021_540_MOESM1_ESM.doc]

**Supplementary Figure 1:The specific modeling method andmodel identification：**

**1.Method**

**Measurement of cell viability by CCK-8 ,cellular morphology and apoptosis assays**

A CCK-8 kit was used to test cell viability following treatment with different compounds in 96-well plates. The cells were segregated into 96-well plates at a density of 2000 cells/ml, and the cell suspension was thoroughly mixed before adding the sample. Cells were then put into a sterile adding tank with a spout to add 100 μl per well at 37°C. After being cultured in a CO2 incubator for 48 h, 0.01 μm, 0.1 μm, 1 μm, 2 μm, 5 μm, and 10 μm Aβ42 were added and left for different durations (3 h, 6 h, 24 h, 48 h, and 72 h). After the corresponding duration, the cells were extracted from the CO2 incubator at 37°C, 100μl CCK-8 dilution (original solution of CCK-8: DMEM = 1:10) was carefully added to the test well and incubated in the CO2 incubator at 37°C for 1 to 4 h. The logarithmic growing cells were inoculated in a 96-well plate at a density of 2000 cells/ml and cultured in a CO2 incubator at 37°C for 48 h.

Then, the SH-SY5Y cells were washed twice with PBS and viewed under an inverted microscope. The morphology of the cells was observed under ×40 magnification. The SH-SY5Y cells were washed twice with PBS and viewed under an inverted microscope. The morphology of the cells was observed under ×40 magnification.

The steps of apoptosis induction were as follows: First, the 10× Binding Buffer was diluted into 1× Binding Buffer by adding double-steamed water; normal trypsin digestion was performed, ensuring that the number of cells in each sample reached 1×10^6^, and the samples were washed with ice-cold PBS 3 times; 1× Binding Buffer 1 ml suspension cells were added, centrifuged at 1000 RPM for 5 min, and the supernatant was dumped; then, 1× Binding Buffer 1 ml resuspended cells; 100 μl cells were added to the flow cytometry test tube; Annexin V-FITC 5 μl was added to each test tube, and after blending, the solution was left to stand at room temperature for 10 min in the dark. We then added 5 μl propidium iodide to each test tube, mixed well, and incubated at room temperature for 5 min away from any light. Finally, we added PBS to 500 μl, mixed gently, then conducted flow cytometry .

**2.Result**

To observe the Aβ_42_‐induced cytotoxicity and protective effects of SZL formula on Aβ_42_-induced neurotoxicity, the cell death, viability release was assessed using the CCK-8 and apoptosis assays. These results suggest that when Aβ_42_ was incubated for 48 h and 72 h, compared with the control group, Aβ_42_ cells cultured at 2 μm, 5 μm, and 10 μm had a lower OD value (*P* < 0.01; Supplementary Fig 1A). Combined with the experimental results of CCK-8, 48 h was selected as the intervention time of aging Aβ_42_, because the OD value of aging Aβ_42_ at 2 μm, 5 μm, and 10 μm was significantly decreased compared with the control group (*P* < 0.01; Supplementary Fig 1B). With the increase of aging Aβ_42_ concentration, the cell body of SH-SY5Y cells gradually atrophied and became round, and the projections became shorter and even disappeared. At the same time, scattered vacuolar structures of different sizes could be seen in some cytoplasm (Supplementary Fig 1B). To further verify the damaging effect of Aβ_42_ on SH-SY5Y cells, cell apoptosis was detected using flow cytometry. The data suggest that Aβ_42_ in 5 μm increased the apoptosis level of SH-SY5Y cells (*P* < 0.01; Supplementary Fig 1C-E). These findings indicate that Aβ_42_ was cultured at 5 μm for 48 h is the best molding opportunity.


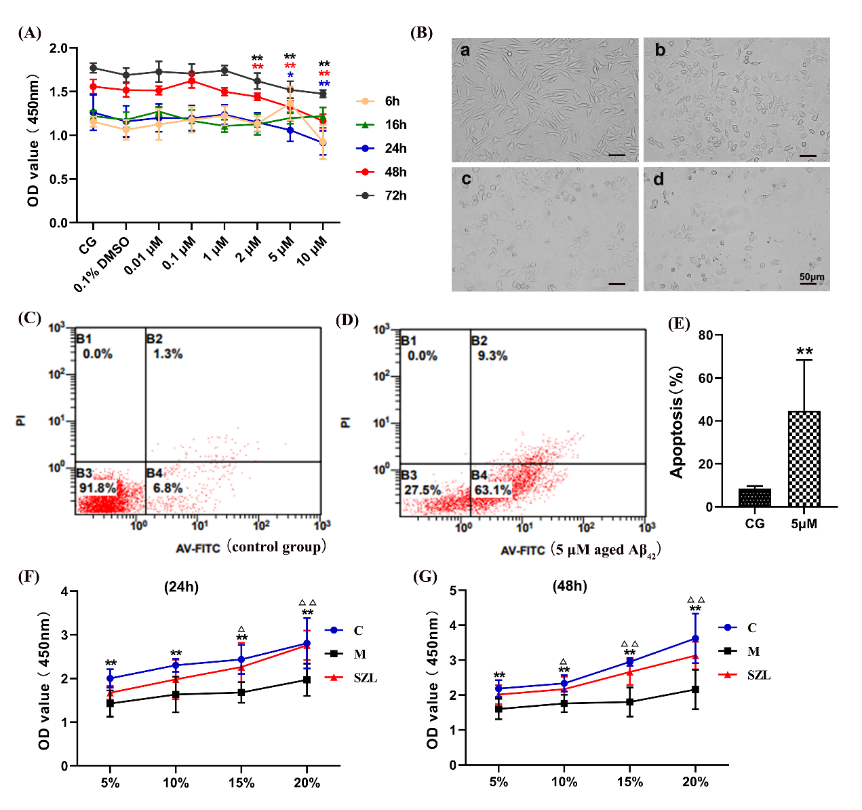


**Supplementary Fig 1:** Aβ_42_-induced SH-SY5Y cell model simulates AD construction in vitro.

The results of flow cytometry scatter plot are as follows. B1 shows mechanically damaged cells, B2 shows inactive cells, i.e. necrotic cells or late apoptotic cells, B3 shows live cells, B4 shows early apoptotic cells, cell apoptosis rate (%) =B2+B4. (A) Time-dose response of SH-SY5Y cells with different concentrations of Aβ_42_, (B) Effect of Aβ42 on morphology of SH-SY5Y cells after 48 h intervention (scale bar=50 μm), (C) apoptosis in control group, (D) apoptosis in 5 μM aged Aβ_42_ cells, (E) apoptosis rate, (F) Time-dose effect of SZL drug serum on SH-SY5Y cells damaged by Aβ_42_. CG: control group. Results are shown as mean ± SD. **P*<0.05, ***P*<0.01 versus Control group, **^△^***P*<0.05, **^△△^***P*<0.01 versus Model group; ANOVA.
