# Supplementary material for: Shen-Zhi-Ling oral liquid ameliorates cerebral glucose metabolism disorder in early AD via insulin signal transduction pathway in vivo and in vitro
Source: Chin Med. 2021 Dec 2;16:128. doi: 10.1186/s13020-021-00540-0 (PMC8638512; doi:10.1186/s13020-021-00540-0)
Supplement: Supplementary file 2 — Additional file 2. Identification of components of SZL into blood. [file 13020_2021_540_MOESM2_ESM.doc]

**Supplementary 2 Identification of components of SZL into blood**

| Name | Formula | Class | mzmed | rtmed | Q1 | Q2 | Q3 |
| --- | --- | --- | --- | --- | --- | --- | --- |
| Methylgallate | C_8_H_8_O_5_ | Phenols | 183.03 | 166.68 | 561223.15 | 0 | 46788746.96 |
| Genkwanin | C_16_H_12_O_5_ | Flavonoids | 283.06 | 598.26 | 304059.56 | 0 | 14318109.91 |
| Sebacic acid | C_10_H_18_O_4_ | Fatty Acyls | 201.11 | 339.18 | 1664063.65 | 0 | 18567817.50 |
| 2-METHYLMALEATE | C_5_H_6_O_4_ | Fatty Acyls | 129.02 | 69.59 | 8931778.33 | 0 | 224326527.55 |
| Glutaric acid | C_5_H_8_O_4_ | Organic acids and derivatives | 131.03 | 96.54 | 11486479.42 | 0 | 152044645.31 |
| Hydroxyferulic acid | C_10_H_10_O_5_ | Phenylpropanoids | 209.04 | 149.27 | 63443956.58 | 0 | 2798859329.34 |
| Cinnamic acid | C_9_H_8_O_2_ | Phenylpropanoids | 147.05 | 149.24 | 930196.60 | 0 | 39290031.14 |
| Naringenin | C_15_H_12_O_5_ | Flavonoids | 271.06 | 465.80 | 2878812.43 | 0 | 30486723.69 |
| Oroxylin A-7-O-beta-D-glucuronide | C_22_H_20_O_11_ | Flavonoids | 459.09 | 431.76 | 2709519.17 | 0 | 20820026.76 |
| Rotenone | C_23_H_22_O_6_ | Flavonoids | 393.13 | 658.35 | 203572.79 | 0 | 73033146.20 |
| 4-Methoxyphenylacetic acid | C_9_H_10_O_3_ | Phenolic acids | 165.06 | 149.25 | 174262583.2 | 0 | 6433287611.12 |
| 1. Sinapic acid | C_11_H_12_O_5_ | Phenylpropanoids | 223.06 | 303.01 | 238227.69 | 0 | 16079415.09 |
| Silibinin | C_25_H_22_O_10_ | Flavonoids | 481.11 | 246.27 | 101402.81 | 0 | 20449164.58 |
| Methylnissolin-3-O-glucoside | C_23_H_12_O_10_ | Flavonoids | 497.12 | 386.73 | 315464.95 | 0 | 54464761.62 |
| Formononetin | C_16_H_12_O_4_ | Flavonoids | 267.07 | 529.93 | 46752882.8 | 0 | 651456456.45 |
| Pinocembrin | C_15_H_12_O_4_ | Flavonoids | 255.07 | 514.22 | 25744683.26 | 0 | 1908207831.67 |
| (+)-Pinoresinol | C_20_H_22_O_6_ | Lignans | 357.14 | 436.68 | 2828957.48 | 0 | 86712838.85 |
| 1. Wogonin | C_16_H_12_O_5_ | Flavonoids | 285.07 | 579.25 | 483124.65 | 0 | 105296741.85 |
| Acetylvanillin | C_10_H_10_O_4_ | Phenols | 195.06 | 316.25 | 30152550.65 | 0 | 105553756.86 |
| Sophocarpine | C_15_H_22_N_2_O | Alkaloids | 247.18 | 92.61 | 16562775.66 | 0 | 2219692181.45 |
| Glyceryl linolenate | C_21_H_36_O_4_ | Miscellaneous | 375.25 | 824.88 | 185414.83 | 0 | 165394462.51 |
| 6-Shogaol | C_17_H_24_O_3_ | Phenols | 277.18 | 703.41 | 953249.52 | 0 | 2766459904.72 |
| Glabrolide | C_30_H_44_O_4_ | Terpenoids | 469.33 | 816.96 | 350908.4504 | 0 | 161088518.40 |
| Cafestol | C_20_H_28_O_3_ | Terpenoids | 317.21 | 778.53 | 1282776.81 | 0 | 47324284.47 |
| Matrine | C_15_H_24_N_2_O | Alkaloids | 249.20 | 88.68 | 13808313.87 | 0 | 758561514.93 |
| Loperamide hydrochloride | C_29_H_34_Cl_2_N_2_O_2_ | Alkaloids | 477.23 | 261.27 | 1383765.117 | 0 | 762417462.70 |
| Paeoniflorin | C_23_H_28_O_11_ | Terpenoids | 498.20 | 275.23 | 1097364.219 | 0 | 3086743247.12 |
| Coumaroyl tyramine | C_17_H_17_NO_3_ | Miscellaneous | 284.13 | 392.94 | 152057.31 | 0 | 39630946.76 |
| Biochanin-7-O-glucoside | C_22_H_22_O_10_ | Flavonoids | 447.13 | 380.29 | 1273633.588 | 0 | 19220028.18 |
| Methyl trans-cinnamic acid | C_10_H_10_O_2_ | Phenylpropanoids | 163.08 | 571.22 | 340704.7738 | 0 | 542044723.24 |
| Tectochrysin | C_16_H_12_O_4_ | Flavonoids | 269.08 | 534.42 | 64083677.19 | 0 | 2431060828.48 |
| Isoalantolactone | C_15_H_20_O_2_ | Terpenoids | 233.15 | 412.47 | 216783.3457 | 0 | 23030015.52 |
| Albiflorin | C_23_H_28_O_11_ | Terpenoids | 481.17 | 250.82 | 1316409.44 | 0 | 8526217753.76 |
| Boldine | C_19_H_21_NO_4_ | Alkaloids | 328.15 | 236.09 | 5739610.111 | 0 | 80656819.54 |
| Sakuranetin | C_16_H_14_O_5_ | Flavonoids | 287.09 | 587.97 | 200173.034 | 0 | 14925636.79 |
| Artemisinic acid | C_15_H_22_O_2_ | Terpenoids | 235.17 | 516.88 | 975079.7412 | 0 | 32214254.97 |
| Perillene | C_10_H_14_O | Terpenoids | 151.11 | 533.45 | 2039405.813 | 0 | 47964486.85 |
| Khellin | C_14_H_12_O_5_ | Flavonoids | 283.06 | 523.94 | 1056910.335 | 0 | 29074780.47 |
| 3,4,5-trimethoxycinnamic acid | C_12_H_14_O_5_ | Phenylpropanoids | 239.09 | 426.08 | 5306956.29 | 0 | 803247657.44 |
| Formononetin | C_16_H_12_O_4_ | Flavonoids | 291.06 | 534.67 | 1539826.064 | 0 | 22958713.29 |
| Isovanillic acid | C_8_H_8_O_4_ | Phenols | 169.05 | 197.00 | 1064290.964 | 0 | 75560939.02 |
| Isorhapontigenin | C_15_H_14_O_4_ | Phenols | 259.10 | 510.00 | 3334618.561 | 0 | 80566020.35 |
| Puerarin | C_21_H_20_O_9_ | Flavonoids | 417.12 | 306.31 | 746802.6796 | 0 | 23928816.35 |
| Propyl gallate | C_10_H_12_O_5_ | Phenols | 213.07 | 316.51 | 35569034.62 | 0 | 124103198.08 |
| Murrayone | C_15_H_14_O_4_ | Coumarins and derivatives | 259.09 | 629.03 | 378954.9793 | 0 | 113723559.12 |
| Pseudojervine | C_33_H_49_NO_8_ | Alkaloids | 588.36 | 305.67 | 747419.9409 | 0 | 66122666.43 |

Note: Q1 peak value of drug-containing serum of SZL; Q2 blank non-drug serum peak; Q3 peak value of SZL oral liquid, all values are reserved two decimal places.
